# Loss of TDP-43 in male germ cells causes meiotic failure and impairs fertility in mice

**DOI:** 10.1016/j.jbc.2021.101231

**Published:** 2021-09-29

**Authors:** Kaitlyn M. Campbell, Yiding Xu, Chintan Patel, Jeremy M. Rayl, Helena D. Zomer, Hari Prasad Osuru, Michael Pratt, Patcharin Pramoonjago, Madeline Timken, Lyndzi M. Miller, Abigail Ralph, Kathryn M. Storey, Yiheng Peng, Jenny Drnevich, Clotilde Lagier-Tourenne, Philip C. Wong, Huanyu Qiao, Prabhakara P. Reddi

**Affiliations:** 1 Department of Comparative Biosciences, University of Illinois Urbana-Champaign, Urbana, Illinois, USA; 2 Department of Pathology, University of Virginia School of Medicine, Charlottesville, Virginia, USA; 3 High-Performance Biological Computing (HPCBio) Group, University of Illinois Urbana-Champaign, Urbana, Illinois, USA; 4 Department of Neurology, Massachusetts General Hospital and Harvard Medical School, Boston, Massachusetts, USA; 5 Department of Pathology, Johns Hopkins School of Medicine, Baltimore, Maryland, USA

**Keywords:** TAR DNA-binding protein of 43 kDa, spermatogenesis, meiosis, male infertility, testis, ADB, antibody dilution buffer, BG, background, BP, biological process, cDNA, complementary DNA, cKO, conditional KO, Crbp2, cellular retinol-binding protein 2, Ct, cycle threshold, DEG, differentially expressed gene, DMC1, dosage suppressor of Mck1 homolog, meiosis-specific homologous recombination (yeast), DSB, double-strand break, FDR, false discovery rate, GO, Gene Ontology, Hormad1, HORMA domain–containing protein 1, Hormad2, HORMA domain–containing protein 2, iCre, improved Cre, IHC, immunohistochemistry, KEGG, Kyoto Encyclopedia of Genes and Genomes, *loxP*, locus of X-over P1, MF, molecular function, NCBI, National Center for Biotechnology Information, NHEJ, nonhomologous end joining, PFA, paraformaldehyde, PL, preleptotene, PND, postnatal day, qRT, quantitative RT, RA, retinoic acid, Rad21L, RAD21 cohesin complex component like 1, RAR, retinoic acid receptor, RBP4, retinol-binding protein 4, Rec8, REC8 meiotic recombination protein, ROI, region of interest, RXR, retinoid X receptor, Sall4a, spalt-like transcription factor 4a, Sall4b, spalt-like transcription factor 4b, SC, synaptonemal complex, Spo11, SPO11 initiator of meiotic double-stranded break, Stra8, stimulated by retinoic acid 8, SYCP2, synaptonemal complex protein 2, SYCP3, synaptonemal complex protein 3, TBST, Tris-buffered saline with 0.1% Tween-20, TDP-43, transactive response DNA-binding protein of 43 kDa

## Abstract

Meiotic arrest is a common cause of human male infertility, but the causes of this arrest are poorly understood. Transactive response DNA-binding protein of 43 kDa (TDP-43) is highly expressed in spermatocytes in the preleptotene and pachytene stages of meiosis. TDP-43 is linked to several human neurodegenerative disorders wherein its nuclear clearance accompanied by cytoplasmic aggregates underlies neurodegeneration. Exploring the functional requirement for TDP-43 for spermatogenesis for the first time, we show here that conditional KO (cKO) of the *Tardbp* gene (encoding TDP-43) in male germ cells of mice leads to reduced testis size, depletion of germ cells, vacuole formation within the seminiferous epithelium, and reduced sperm production. Fertility trials also indicated severe subfertility. Spermatocytes of cKO mice showed failure to complete prophase I of meiosis with arrest at the midpachytene stage. Staining of synaptonemal complex protein 3 and γH2AX, markers of the meiotic synaptonemal complex and DNA damage, respectively, and super illumination microscopy revealed nonhomologous pairing and synapsis defects. Quantitative RT–PCR showed reduction in the expression of genes critical for prophase I of meiosis, including *Spo11* (initiator of meiotic double-stranded breaks), *Rec8* (meiotic recombination protein), and *Rad21L* (RAD21-like, cohesin complex component), as well as those involved in the retinoic acid pathway critical for entry into meiosis. RNA-Seq showed 1036 upregulated and 1638 downregulated genes (false discovery rate <0.05) in the *Tardbp* cKO testis, impacting meiosis pathways. Our work reveals a crucial role for TDP-43 in male meiosis and suggests that some forms of meiotic arrest seen in infertile men may result from the loss of function of TDP-43.

Transactive response DNA-binding protein of 43 kDa (TDP-43) is a ubiquitously expressed and evolutionarily conserved DNA/RNA-binding protein with varied functions, including gene transcription, mRNA splicing, exon skipping, as well as micro-RNA biogenesis (1, 2). TDP-43 contains two RNA recognition motifs with which it binds to DNA and RNA (3). The C-terminal region contains a glycine-rich domain. The very first report on TDP-43 showed that it functions as a sequence-specific transcriptional repressor in human cells (1). A subsequent study demonstrated a role for TDP-43 in exon skipping in mammalian cells (4). TDP-43 has been shown to interact with heterogeneous nuclear ribonucleoprotein proteins to mediate alternative splicing (5, 6). The C-terminal part of TDP-43 also consists of a prion-like intrinsically disordered domain involved in forming protein aggregates (7, 8). Germline deletion of TDP-43 in mice proved to be embryonic lethal indicating the essential nature of the protein (9, 10).

Although TDP-43 became widely known as a protein involved in the pathology of a number of neurodegenerative diseases following the publication by Neumann *et al.*, in 2006 (11), prior to that, we reported cloning of TDP-43 from a mouse testis complementary DNA (cDNA) library as a transcription factor binding to the promoter of the testis-specific *Acrv1* gene (12). The mouse *Acrv1* gene is expressed exclusively in round spermatids, and its promoter contains two TGTGTG motifs—canonical TDP-43-binding sites to which TDP-43 binds *in vitro*. Using transgenic mice as a reporter system, we showed that mutation of the two TGTGTG motifs within the Acrv1 promoter caused premature transcription of a reporter gene in spermatocytes, whereas the WT promoter maintained round spermatid-specific expression *in vivo* (12). This suggested that TDP-43 might repress the *Acrv1* gene expression in spermatocytes *in vivo*. Using the Gal4 reporter assay, we showed that TDP-43 represses gene transcription (13). In addition, we also demonstrated a role for TDP-43 in the insulator function required to keep the *Acrv1* gene silent in the somatic tissues (14). Chromatin immunoprecipitation showed occupancy of TDP-43 at the promoter of the *Acrv1* gene, both in spermatocytes as well as round spermatids. Interestingly, chromatin immunoprecipitation also showed that RNA pol II and the pol II pause machinery were loaded on the Acrv1 promoter in spermatocytes prior to the expression of Acrv1 mRNA in round spermatids (13). Taken together, our previous work showed that TDP-43 functions as a transcriptional repressor and that *Acrv1* is a TDP-43 target gene *in vivo*. Although our studies mainly focused on its role as a transcriptional repressor of the *Acrv1* gene thus far, we anticipate that TDP-43 plays a global role in the regulation of gene expression in the testis, both at the transcriptional as well as post-transcriptional level.

Immunolocalization studies of the mouse testis showed that TDP-43 expression begins in the intermediate and type B spermatogonia, peaks in preleptotene (PL) spermatocytes, and remains high in pachytene spermatocytes (15). The round spermatids express TDP-43, but the expression gradually tapers off in late-stage spermatids. In addition to germ cells, Sertoli cells also express TDP-43. The location of TDP-43 was nuclear in all the aforementioned cells (15). The pattern of spatiotemporal expression of TDP-43 within the seminiferous epithelium (highest expression seen in PL and pachytene spermatocytes) indicates a functional role for the protein in male germ cell differentiation and sperm formation, particularly during meiosis. In support of this, TDP-43 was found to be aberrantly expressed in testicular germ cells and spermatozoa of some infertile men (16). On the basis of the aforementioned data, we hypothesized that TDP-43 would be essential for spermatogenesis and male fertility. To test, we have generated conditional KO (cKO) mice lacking *Tardbp* in adult male germ cells.

Our experimental results show that in the absence of TDP-43, spermatocytes were unable to complete prophase I of meiosis leading to maturation arrest. Male mice bearing germ cell KO of *Tardbp* produced fewer and morphologically abnormal sperm. *Tardbp* KO male mice were severely subfertile. Consistent with its role as a multifunctional protein, loss of TDP-43 resulted in global changes in the expression of genes in the testis: 1036 genes were upregulated and 1638 were downregulated.

## Results

### Loss of TDP-43 leads to arrest of spermatogenesis

**Figure 1:**
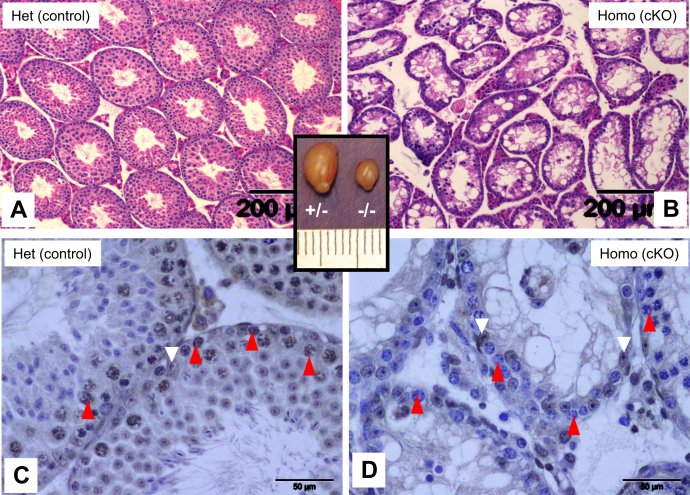
**Testis phenotype of *Tardbp* cKO mice.** Testis cross sections of heterozygous controls (*A* and *C*) and homozygous cKO mice (*B* and *D*) PND35 mice showing that loss of TDP-43 leads to atrophy of seminiferous tubules, germ cell loss, and vacuole formation (*B*). Immunohistochemistry shows TDP-43 expression in germ cells (*red arrowheads*) and Sertoli cells (*white arrowheads*) of control mice (panel *C*) and lack of TDP-43 in germ cells (*red arrowheads* of panel *D*) of cKO testis but not in Sertoli cells (*white arrowheads*), thus confirming germ cell–specific KO of TDP-43. Inset shows the size of the testis of control (+/−) *versus* cKO (−/−) mice. cKO, conditional KO; PND35, postnatal day 35; TDP-43, transactive response DNA-binding protein of 43 kDa.

In order to investigate the functional requirement of TDP-43 for spermatogenesis in mice, we crossed floxed TDP-43 mice with stimulated by retinoic acid (RA) gene 8 (Stra8)–improved Cre (iCre) deleter mice to delete the TDP-43 gene in the spermatogonial stage of male germ cell differentiation. In floxed TDP-43 mice, exon 3 of *Tardbp* (gene symbol for TDP-43), which codes for two critical RNA recognition motifs, is flanked by the *loxP* (locus of X-over P1) sites. Previous studies using these mice showed that Cre-mediated excision leads to loss of TDP-43 protein in target tissues (10). Stra8–iCre–mediated excision of floxed genes is specific for the male germ cells and begins by postnatal day 4 (PND4) within the undifferentiated spermatogonia of the testis (17, 18). TDP-43 protein first appears in intermediate and type B spermatogonia ((15) and Fig. S1). Thus, Stra8–iCre–mediated excision is expected to lead to loss of TDP-43 in intermediate spermatogonia and all subsequent male germ cell types. To assess the phenotypic effect, we analyzed male mice with the genotype *Tardbp*^Flox/null^, Stra8–Cre+ (referred to as homozygous cKO). The heterozygous *Tardbp* ^Flox/wt^ Stra8–Cre+ mice served as control (het control). We first analyzed the testes of PND35 males, the time point at which the first wave of spermatogenesis will have completed. Testis size was severely reduced in cKO mice compared with control littermates (Fig. 1, *inset*). In cross section, the diameter of the seminiferous tubules appeared narrower compared with control (Fig. 1, *A* and *B*). Histological examination showed extensive germ cell depletion and the presence of vacuoles within the tubules. The control (heterozygous) testes showed germ cells at all stages of differentiation including spermatozoa at the luminal interface indicating proper completion of the first round of spermatogenesis (Fig. 1*A*). It must be noted here that there was no difference between the heterozygous and WT mice in terms of TDP-43 protein levels (Fig. S1*B*) or testis phenotype. The cKO testes, however, showed fewer differentiating cell types in the epithelium and the absence of spermatozoa (Fig. 1*B*). Immunohistochemistry (IHC) was performed using anti-TDP43 antibody to verify the status of TDP-43 expression. Control mice expressed TDP-43 in germ cells as well as Sertoli cells (Fig. 1*C*), whereas the cKO testis showed TDP-43 only in Sertoli cells (Fig. 1*D*), thus confirming KO of *Tardbp* within the spermatogonia and all subsequent germ cell types. At higher magnification, spermatogonia and a few meiotic cells could be seen in cKO testis but no round spermatids (Fig. 1*D*). Thus, absence of round spermatids at PND35 suggested failure to complete meiosis in *Tardbp* cKO mice.

**Figure 2:**
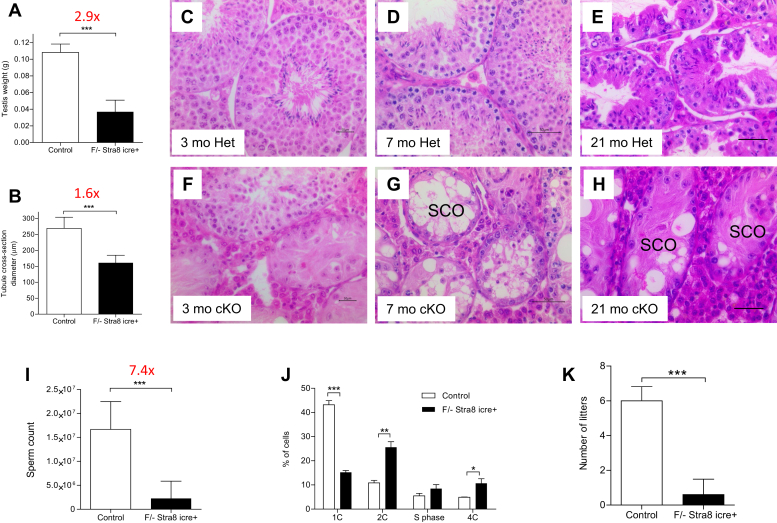
***Tardbp* cKO male mice were severely subfertile.** Decline in testis weight (*A*) and the diameter of seminiferous tubules (*B*) in 3-month-old cKO mice compared with heterozygous control. H&E-stained cross sections of testes from 3-, 7-, and 21-month-old control (*C*–*E*) and cKO (*F*–*H*) depicting pathology in cKO mice over time. Note the Sertoli cell only (SCO)–like syndrome in panels *G* and *H*. The cKO mice produced 7.4 times less number of caudal sperm compared with controls (*I*). Flow cytometry data showing a significant difference in the percentages of 1C, 2C, and 4C cells (*J*) between the testicular cells of cKO (*dark bars*) *versus* control mice (*white bars*). Fertility trial (*K*) showed that the cKO males (*dark bar*) produced a significantly less number of litters compared with control males (*white bar*) over a 6-month period. The scale bar for images *C*–*H* represents 50 μm. cKO, conditional KO.

We then extended the analysis to older mice ranging from 2 to 21 months of age (n = 30). Overall, we observed a 2.9-fold decrease in testis weight (*p* < 0.0001) and 1.6-fold reduction in the diameter of the seminiferous tubule (*p* < 0.0001) (Fig. 2, *A* and *B*). Testes of cKO mice at 3, 7, and 21 months of age showed progressively worse depletion of germ cells and vacuole formation within the seminiferous tubules (Fig. 2, *C*–*H*). At advanced ages, the seminiferous epithelium only consisted of Sertoli cells, reminiscent of the human infertility condition known as Sertoli cell–only syndrome (Fig. 2, *G* and *H*). IHC using antibody to the Sertoli marker Sox 9 confirmed that the cells remaining within the seminiferous epithelium of the cKO mice are in fact Sertoli cells (Fig. S2, *A* and *B*).

We observed a variation in the penetrance of the phenotype in terms of germ cell loss in cKO mice (n = 30; data not shown). A majority of the cKO mice showed severe germ cell depletion with few spermatozoa, whereas some cKO males showed progression of spermatogenesis in a portion of seminiferous tubule cross sections. Consistent with this, the total number of caudal sperm varied averaging at 2.2 million per cKO mouse compared with 16 million per control mouse (Fig. 2*I*). We quantified the different types of germ cells in cKO mice testes by flow cytometry using testicular cells isolated from mice aged 3 to 4 months (Fig. 2*J*). Compared with WT controls, cKO mice testes showed a statistically significant decrease in 1 N cells (round spermatids) and an increase in 2 N (spermatogonia, Sertoli, peritubular, and Leydig cells) and 4 N (primary spermatocytes) cells. Accumulation of 2 N and 4 N cells in *Tardbp* cKO testes indicated arrest in meiosis, mimicking the meiotic arrest phenotype seen in biopsies obtained from some azoospermic infertile men.

### Loss of TDP-43 in testicular germ cells leads to subfertility

Majority of spermatozoa collected from cauda epididymides of *Tardbp* cKO mice were morphologically abnormal. Sperm with deformed or detached head accounted for roughly half of the population. Approximately 55% of the cKO sperm showed head and midpiece bent over the principal piece of the tail (Fig. S3, *A*–*E*). To address the requirement of TDP-43 for fertility in male mice, we conducted fertility trials. Three-month-old *Tardbp*^Flox/null^ Stra8–Cre+ males were cohabited with age-matched WT females. A total of four separate cKO males were used in the fertility trial. Breeding pairs of 3-month-old WT males (n = 5) and females served as controls. At the end of the 6-month fertility trial period, control mice gave birth to an average of 6 litters, whereas the cKO mice produced an average of 0.6 litters during the same period (Fig. 2*K*). Thus, TDP-43 deletion in male germ cells caused a significant reduction in male fertility in mice (*p* < 0.0001).

### At what point of spermatogenesis is TDP-43 critical?

Next, we wanted to systematically examine at what point spermatogenesis was halted because of the lack of TDP-43 in spermatogonia. Since TDP-43 expression was not detected in undifferentiated spermatogonia (15), we did not expect to see defects in early stage spermatogonia. TDP-43 expression, however, begins in the intermediate spermatogonia of WT mice. Therefore, depletion of TDP-43 could potentially affect the progression of type B spermatogonia to PL spermatocytes. To address this, we explored the expression of Stra8 as a marker of PL spermatocytes. IHC showed intact PL spermatocytes expressing Stra8 in cKO mice (Fig. S2, *C* and *D*). Stra8 expression in cKO mouse PL spermatocytes also indicated that the RA signaling pathway for entry into meiosis occurred normally up to that point.

### TDP-43 is required for the completion of prophase I of meiosis

**Figure 3:**
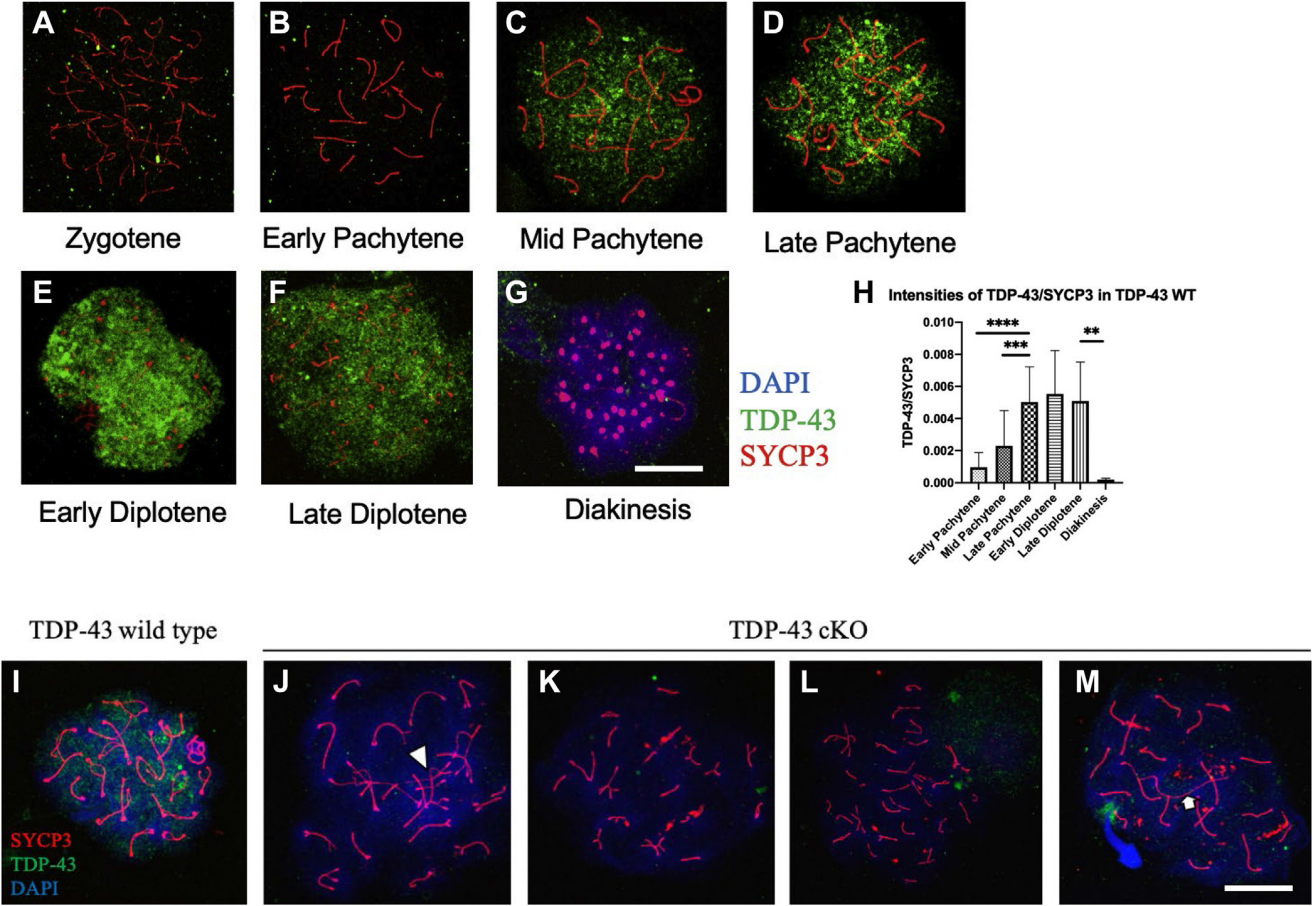
**Dynamics of TDP-43 expression during prophase I of meiosis.** Immunofluorescence of meiotic chromosome spreads from WT mice showing the dynamics of TDP-43 expression during prophase I of meiosis. Antibodies to TDP-43 (*green*) and SYCP3 (*red*) were used; nuclear counterstaining was done with DAPI (*blue*). TDP-43 is predominantly expressed between midpachytene to late diplotene stages of prophase I (*C*–*F*). Very little TDP-43 expression was found in zygotene, early pachytene, and diakinesis stages (*A*, *B*, and *G*, respectively). Quantification of TDP-43 expression is shown in panel *H*. Panel *I* shows a diplotene spermatocyte of WT mouse stained with antibodies to TDP-43, SYCP3, and DAPI. The cKO mice showed no expression of TDP-43 and various types of defects occurring at pachytene-like (*K*–*M*) and diplotene-like (*J*) spermatocytes. *Arrowhead* in (*J*) indicates synapsis partner exchange, and the *arrow* in (*M*) indicates an extra-long chromosome. The scale bar represents 10 μm. cKO, conditional KO; DAPI, 4′,6-diamidino-2-phenylindole; SYCP3, transactive response DNA-binding protein of 43 kDa; TDP-43, transactive response DNA-binding protein of 43 kDa.

Prophase I of meiosis consists of five stages, including leptonema, zygonema, pachynema, diplonema, and diakinesis. We performed immunolabeling with TDP-43 and synaptonemal complex (SC) protein 3 (SYCP3) on WT mouse testis meiotic chromosome spreads to determine the dynamics of expression of TDP-43 during meiotic prophase I (Fig. 3, *A*–*G*). TDP-43 was not found until the midpachytene stage (Fig. 3*C*). This was consistent with earlier findings in which IHC showed lack of TDP-43 expression in leptotene and zygotene spermatocytes ((15) and Fig. S1). TDP-43 expression started in midpachytene spermatocytes, peaked in late pachytene, and remained high until the diplotene spermatocyte stage, and nearly eliminated in diakinesis (Fig. 3, *C*–*G*). Next, we quantified TDP-43 expression during meiotic prophase I. TDP-43 fluorescent signal was normalized with that of the lateral element protein SYCP3 and plotted. In agreement with the immunofluorescence images, the highest expression of TDP-43 was seen from late pachytene to late diplotene stages (Fig. 3*H*), indicating that TDP-43 might play an important role in the meiotic processes occurring in pachytene and diplotene spermatocytes. Consistent with this, immunofluorescence of meiotic chromosome spreads from the cKO testis showed cells arrested mostly at midpachytene stage, whereas some progressed to later stages of prophase I (Fig. 3). SYCP3 staining showed various synapsis defects in the spermatocytes of cKO mice. Compared with the WT spermatocytes (Fig. 3*I*), diplotene-like spermatocytes from *Tardbp* cKO showed nonhomologous chromosome synapsis (*arrowhead*) (Fig. 3*J*). Zygotene-like spermatocytes from *Tardbp* cKO showed nonhomologous chromosome synapsis, short chromosome length, and abnormal number of chromosomes (n = 17 in *K*, n = 25 in *L* of Fig. 3). The abnormal numbers of chromosomes in cKO testes may have resulted from abnormal synapsis between nonhomologous chromosomes. Some pachytene-like spermatocytes showed abnormally long chromosomes (*white arrow* in Fig. 3*M*). The data indicated that loss of TDP-43 in male germ cells disrupted critical processes such as homologous chromosome pairing and recombination that occur at pachynema during prophase I of meiosis.

**Figure 4:**
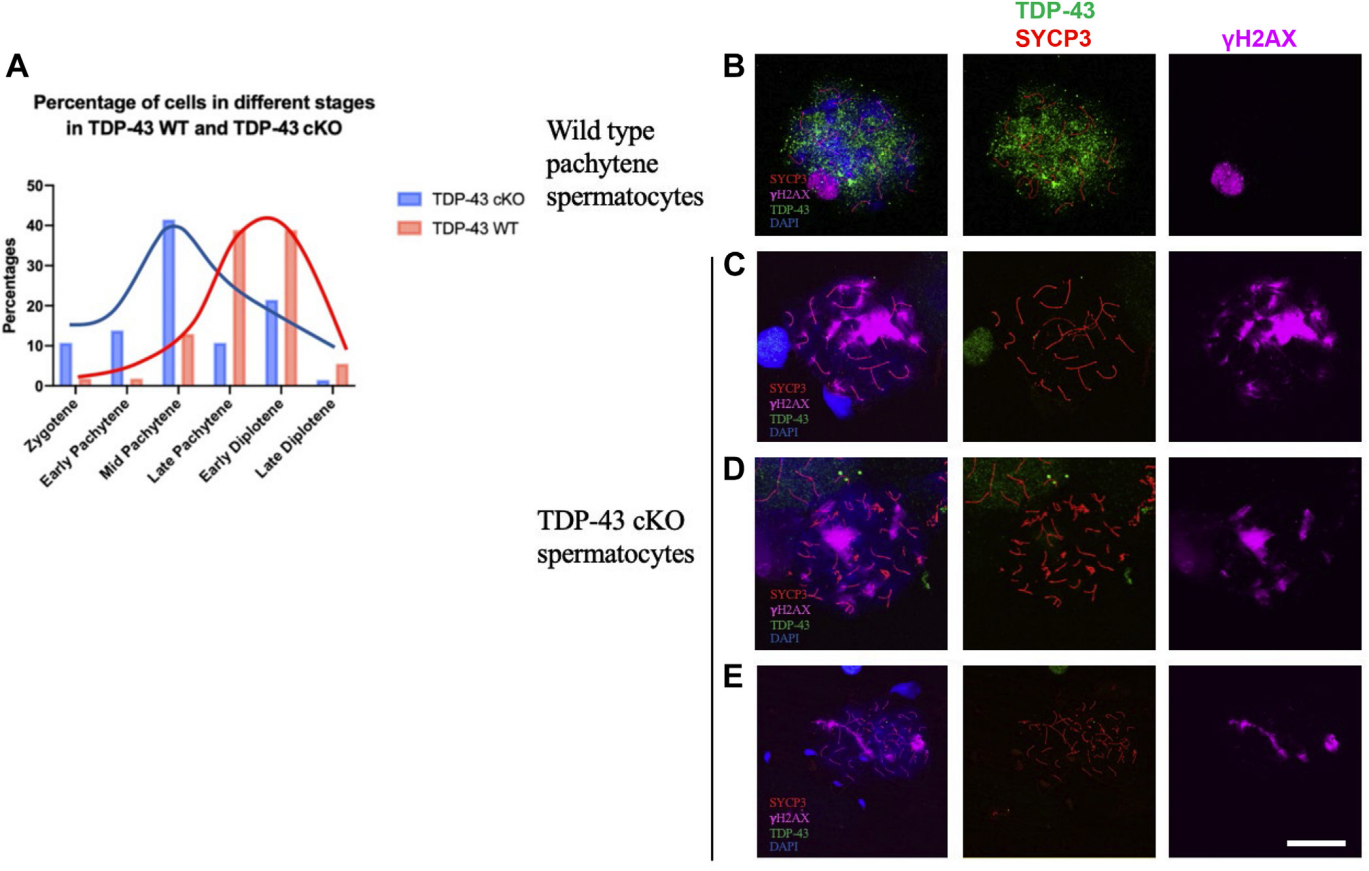
**Loss of TDP-43 causes meiotic failure in cKO mice.***A*, percentage of cells at different meiotic stages in TDP-43 WT (*orange*) and conditional knockdown mutant (*blue*). Most spermatocytes are at late pachytene or early diplotene stage in WT, but accumulation at midpachynema stage occurred in *Tardbp* cKO. *B*–*E*, *Tardbp* cKO spermatocytes with extensive γH2AX showing nonhomologous chromosome pairing. *B*, WT pachytene spermatocytes. γH2AX staining covers the sex body. *C*–*E*, γH2AX persists in pachytene-like spermatocytes with synapsis defects from *Tardbp* cKO. γH2AX staining represents nonhomologous synapsed regions; γH2AX foci/flares on autosomes likely indicate unrepaired DSBs. The scale bar represents 10 μm. cKO, conditional KO; DSB, double-strand break; TDP-43, transactive response DNA-binding protein of 43 kDa.

Next, we compared the number of cells at various stages of prophase I in the WT *versus* cKO testis to determine the stage at which meiotic arrest occurred in cKO mice (Fig. 4*A*). *Tardbp* cKO mice showed accumulation of cells in zygotene, early pachytene, and mostly at midpachytene stages. *Tardbp* cKO mice showed a decline in the number of late pachytene and diplotene spermatocytes, indicating failure to advance to these stages in the absence of TDP-43. This suggested that TDP-43 is required for meiotic processes that occur during pachynema.

To further characterize the meiotic defects, meiotic chromosome spreads were simultaneously stained with antibodies to γH2AX, SYCP3, and TDP-43 (Fig. 4, *B*–*E*). In WT pachytene spermatocytes, γH2AX staining is restricted to the sex-body regions (Fig. 4*B*, *right panel*). This is because the X and Y chromosomes pair at the pseudoautosomal region and the remaining unpaired X and Y chromosomal regions, which are transcriptionally silenced, are stained with γH2AX. In contrast, extensive and persistent γH2AX staining, pseudosex bodies, was seen over the autosomes in *Tardbp* cKO spermatocytes (Fig. 4, *C*–*E*, *right panels*). These pseudosex bodies are indicative of synapsis problems including nonhomologous chromosome pairing consistent with the abnormal numbers of chromosomes in meiotic spreads (Fig. 3, *K* and *L*). Furthermore, retention of γH2AX foci/flares on autosomes in cKO testis could also indicate a delay in the repair of DNA double-strand breaks (DSBs). IHC of testis cross sections further confirmed that the nuclei of cKO spermatocytes show extensive γ-H2AX staining (Fig. S4, *A* and *B*). Consistent with failure to complete meiosis, several spermatocytes are seen undergoing apoptosis as revealed by TUNEL staining (Fig. S4, *C*–*E*).

**Figure 5:**
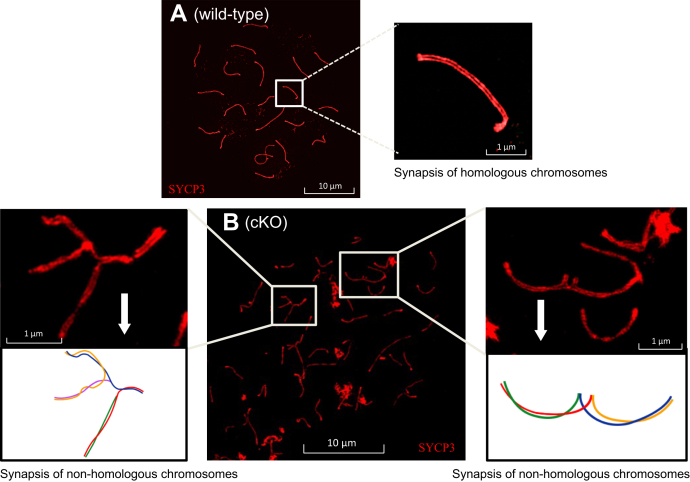
**Loss of TDP-43 causes synapsis defects in spermatocytes.** Nonhomologous pairing and defective synapsis revealed by SYCP3 staining and structure illumination microscopy (SIM) microscopy. *A*, normal homologous pairing and synapsis in WT spermatocyte at pachynema. Two parallel SYCP3 lines in the inset indicate end-to-end synapsis of a pair of homologous chromosomes. *B*, various defective synapsis patterns in *Tardbp* cKO are shown. The schematics of the *insets* demonstrate partner exchange among nonhomologous chromosomes in *Tardbp* cKO. Note: five chromosomes participate in partner exchange in the *left inset* and four in the *right inset*. The scale bar represents 1 μm in panels *A* and *B* and 10 μm in the magnified images. SYCP3, synaptonemal complex protein 3; TDP-43, transactive response DNA-binding protein of 43 kDa.

We performed structure illumination microscopy microscopy to obtain higher resolution images of homologous chromosome pairing and synapsis in WT and cKO spermatocytes. SYCP3 is a marker for the meiotic chromosome axes. WT spermatocytes at pachynema showed two parallel SYCP3 lines indicating proper synapsis between homologous chromosomes (Fig. 5*A*). The inset in Figure 5*A* shows the end-to-end pairing of homologous chromosomes in WT mice. In contrast, *Tardbp* cKO spermatocytes showed synapsis partner exchange between nonhomologous chromosomes (Fig. 5*B*). Examples of defective synapsis patterns are shown in insets of Figure 5*B*. Partner exchange between five and four different nonhomologous chromosomes is depicted in the schematics accompanying the insets on the left and right sides of Figure 5*B*, respectively. This showed that loss of TDP-43 causes nonhomologous chromosome synapsis in pachytene spermatocytes. Collectively, these data suggest that TDP-43 is required for normal synapsis of chromosomes in mouse spermatocytes.

### Dysregulation of genes in the RA pathway and meiosis

**Figure 6:**
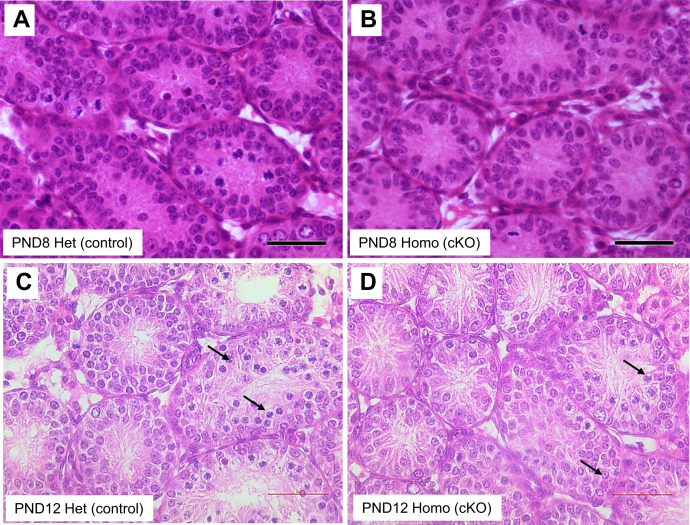
**Onset of testis pathology in *Tardbp* cKO.** H&E images of testis cross sections of prepubertal mice showing the onset of phenotypic changes. At PND8, there was no difference in the cellularity of the seminiferous epithelium between the control (*A*) and cKO mice (*B*). At PND12, the cKO testis shows the onset of pathology with decreased cellularity (*D*) compared with the control (*C*). Note the difference in the appearance of spermatocytes shown by *arrows* in panels *C* and *D*. The scale bar represents 50 microns. cKO, conditional KO; PND8, postnatal day 8; PND12, postnatal day 12.

**Figure 7:**
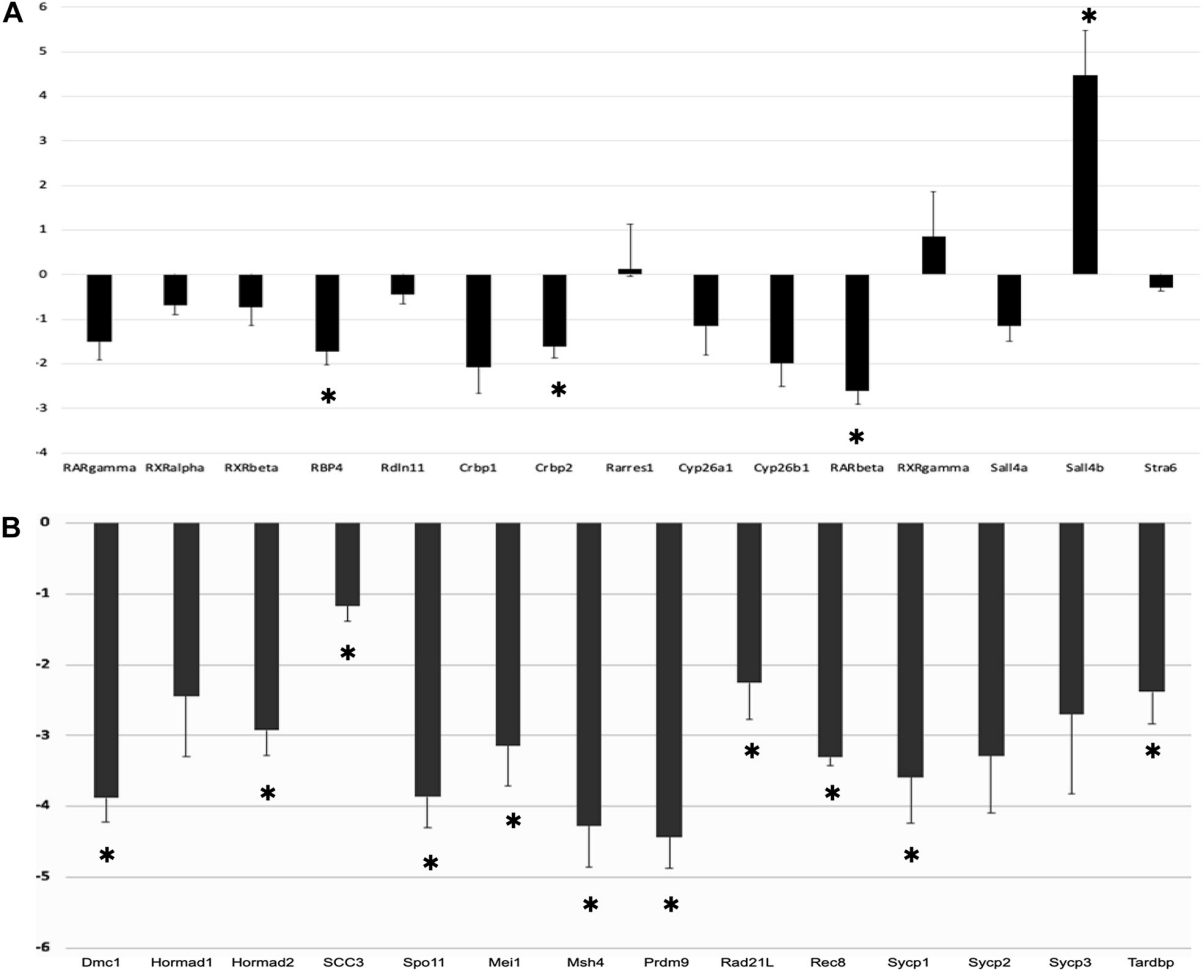
**Dysregulation of gene expression in *Tardbp* cKO testis.** Quantitative RT–PCR of genes involved in the retinoic acid pathway (*A*) and meiosis (*B*). Average log_2_ fold change in mRNA transcription of genes for three biological replicates of postnatal day 12 (PND12) cKO mice compared with WT as no change (log_2_ fold change = 0). In panel *A*, RBP4, Crbp2, RAR beta, and Sall4b showed significant difference (*p* < 0.05). From the gene set in panel *B*, Mei1, Prdm9, Spo11, DMC1, Rad21L, Rec8, Hormad2, SYCP1, and MSH4 showed statistically significant differences (*p* < 0.05). cKO, conditional KO; Crbp2, cellular retinol-binding protein 2; DMC1, dosage suppressor of Mck1 homolog, meiosis-specific homologous recombination (yeast); Hormad2, HORMA domain–containing protein 2; Mei1, meiotic double-stranded break formation protein 1; MSH4, MutS homolog 4; Prdm9, PR/SET domain 9; Rad21L, RAD21 cohesin complex component like 1; RAR, retinoic acid receptor; RBP4, retinol-binding protein 4; Rec8, REC8 meiotic recombination protein; Sall4b, spalt-like transcription factor 4b; Spo11, SPO11 initiator of meiotic double-stranded break; SYCP1, synaptonemal complex protein 1.

Since TDP-43 is a transcription factor/RNA binding protein, we asked if the loss of TDP-43 altered the expression of candidate genes known to be involved in the initiation and progression of meiosis. Evaluation of the cKO testis in adult mice showed that over time there was pronounced atrophy of the seminiferous epithelium. Therefore, we reasoned that in order to observe direct effects of loss of TDP-43, it would be more appropriate to probe for differences in gene expression at the time of the initial onset of pathology in cKO testis. To determine the time point at which phenotypic changes have begun in the testis of the KO mice, we examined the histology of the testis at prepubertal ages. Histologically, there was no difference between the control and cKO testis at PND8 (Fig. 6, *A* and *B*). By PND12; however, there were noticeable changes in the cellularity of the seminiferous epithelium and the appearance of spermatocytes in the cKO testis (Fig. 6, *C* and *D*). The severity of pathology became progressively worse at PND15 (data not shown). The period between PND8 and PND12 coincides with the onset of prophase I of meiosis in the testis under the control of RA signaling. Based on a previous publication of RA pathway genes in the testis (19), we analyzed the expression of retinoic acid receptor (RAR) beta, RAR gamma, retinoid X receptor (RXR) alpha, RXR beta, RXR gamma, retinol-binding protein 4 (RBP4), Rdln11, cellular retinol-binding protein 1, cellular retinol-binding protein 2 (Crbp2), retinoic acid receptor responder 1, cytochrome P450 26A1, spalt-like transcription factor 4a (Sall4a), spalt-like transcription factor 4b (Sall4b), and signaling receptor and transporter of retinol STRA6 at PND12. Quantitative RT (qRT)–PCR showed that RBP4, Crbp2, RAR beta, and Sall4b showed significant differences (*p* < 0.05). RBP4, Crbp2, and RAR beta showed decreased gene expression in *Tardbp* cKO testes, whereas Sall4b showed a significantly high level of expression (Fig. 7*A*). This suggested that TDP-43 may functionally impact the execution of RA signaling for the initiation and maintenance of meiosis in spermatocytes. Next, to investigate the effect of TDP-43 on the expression of genes regulating prophase I of meiosis, we analyzed the expression of genes involved in DSB formation, synapsis, and recombination, including *Mei1* (meiotic double-stranded break formation protein 1), *Prdm9* (PR/SET domain 9), *Spo11* (initiator of meiotic double-stranded breaks), *Dmc1* (DNA meiotic recombinase 1), *Rad21L* (RAD21 cohesin complex component like 1), *Rec8* (meiotic recombination protein REC8), *Hormad1* (HORMA domain-containing protein 1), *Hormad2* (HORMA domain-containing protein 2), *Sycp1*, (synaptonemal complex protein 1), *Sycp2* (synaptonemal complex protein 2) *Sycp3* (synaptonemal complex protein 3), and *Msh4* (MutS homolog 4). All these (with the exception of *Hormad1*, *Sycp2*, *and* *Sycp3*) showed a statistically significant decrease in gene expression in PND12 cKO testes (Fig. 7*B*). Finally, qRT–PCR confirmed conditional deletion of the *Tardbp* gene and showed 2.3-fold reduction in the expression of *Tardbp* mRNA (*p* < 0.05) in the cKO testes compared with controls (Fig. 7*B*). The data suggest that loss of TDP-43 in testicular germ cells had an impact on the mRNA expression of candidate genes critical for prophase I of meiosis.

### Global gene expression changes in TDP-43 KO testis

**Figure 8:**
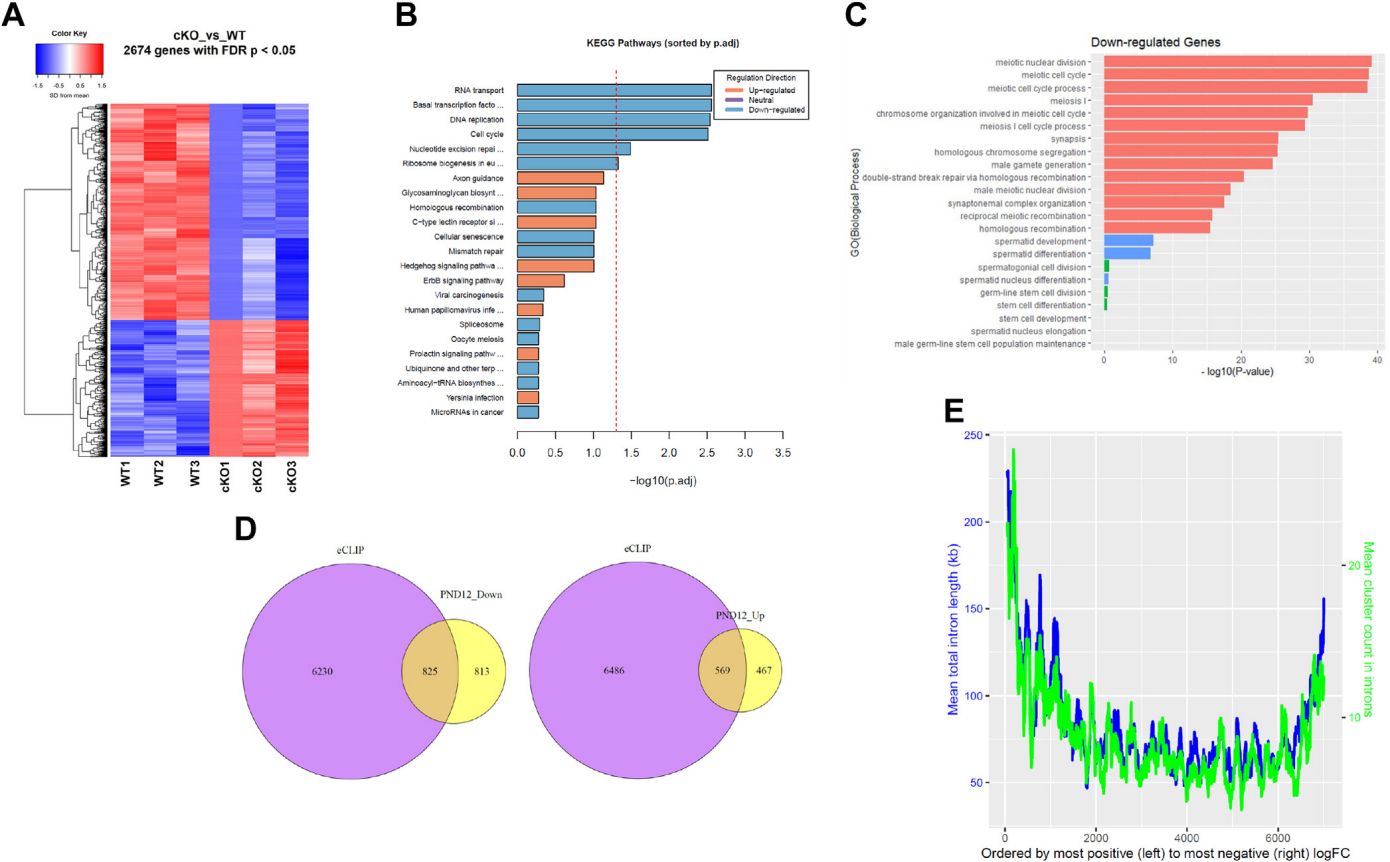
**RNA-Seq and bioinformatics analysis.** RNA-Seq to identify differentially expressed genes (DEGs) in *Tardbp* cKO testis (n = 3) and bioinformatics analysis to identify overlap with previously identified TDP-43 binding RNAs. *A*, heat map showing 2674 DEGs with 1036 upregulated and 1638 downregulated genes (FDR < 0.05). *B*, KEGG pathway analysis shows top-enriched pathways for upregulated and downregulated DEGs. *C*, top-enriched GO biological processes for upregulated and downregulated genes. *D*, Venn diagram showing comparison of testis DEGs with genes previously identified to have TDP-43 binding sites (CLIP). Approximately 50% of upregulated and 54% of downregulated DEGs contained TDP-43 binding sites. *E*, correlation between *Tardbp* cKO testis RNA-Seq and previously published brain TDP-43 CLIP-Seq data. Genes were ranked based on their degree of expression in *Tardbp* cKO testis (*x*-axis). The mean total intron length for the next 100 genes was plotted (*y*-axis, *blue line*). Likewise, the mean number of intronic CLIP clusters found in the next 100 genes from the ranked list were plotted (*y*-axis, *green line*). cKO, conditional KO; FDR, false discovery rate; GO, Gene Ontology; KEGG, Kyoto Encyclopedia of Genes and genomes; TDP-43, transactive response DNA-binding protein of 43 kDa.

Next, in order to identify global gene expression changes caused by the loss of TDP-43 in the testis, we performed RNA-Seq using testes from PND12 WT (control) and *Tardbp* cKO (experimental) mice (n = 3 each). PND12 was chosen as a time point because at this age overt phenotypical changes of the seminiferous epithelium have not yet set in, thus making it possible to identify direct target genes of TDP-43. A total of 2674 genes were differentially expressed (false discovery rate [FDR] <0.05) in the cKO testis compared with controls. Of these, 1036 genes were upregulated and 1638 genes were downregulated (Fig. 8*A*). We generated a heat map of the top 80 protein-coding differentially expressed genes (DEGs), which showed 21 upregulated and 59 downregulated genes (Fig. S5*A*). Thus, overall, there were more downregulated than upregulated genes in this early phase of TDP-43 pathology in mouse testis. To gain clues on the underlying causes of testis pathology upon loss of TDP-43, we performed functional classification of the DEGs using Kyoto Encyclopedia of Genes and Genomes (KEGG) pathway enrichment analysis. RNA transport, cell cycle, DNA replication, and basal transcription factor pathways had more DEG genes than expected (FDR-adjusted *p* value < 0.05), and they primarily were downregulated (Fig. 8*B*). The top pathways with primarily upregulated genes were axon guidance and other signaling pathways although these did not quite meet the threshold for more genes than expected (0.05 < FDR *p* value < 0.1). Next, we performed Gene Ontology (GO) enrichment analysis. Genes that were differentially expressed in the *Tardbp* cKO testis were associated with a number of biological processes (BPs) and molecular functions (MFs). The top two BPs for downregulated genes were cell cycle and cellular metabolic process (Fig. S5*B*). Genes known to be involved in meiosis were among these including Hormad2, Sycp2, Syep3, Msh4, Msh5, Rad21L, Rpa2, and Meiob. Upregulated genes including, Tead1, Nrbp2, Erbb4, Nav2, and Mpzl2, were associated with several BPs related to development (Fig. S5*C*). In terms of MF, nucleic acid–binding function (Polr2b, Taf4b, Smc4, Hmgb2, Rbmy, and Ythdc2) was among the top four for the downregulated genes (Fig. S6*A*). Protein binding and DNA binding were enriched for upregulated genes (including Creb5, Tcf7l2, Six5, Foxo6, Foxp4, and WT1; Fig. S6*B*). Thus, the unbiased approach showed that loss of DNA-/RNA-binding protein TDP-43 leads to dysregulation of gene expression early in the disease process (PND12), which might be the precursor for the subsequent phenotypic alterations to male germ cells and disruption of spermatogenesis. Next, we asked if the differentially regulated genes affected the BPs associated with spermatogenesis. Consistent with the observed phenotype of the cKO testis, we found that multiple processes central to the execution of meiosis, including meiotic cell cycle, synapsis, homologous chromosome segregation, and homologous recombination, were significantly enriched for downregulated genes (Fig. 8*C*).

### Overlap between DEGs in testis and known TDP-43 binding RNAs

TDP-43 contains two canonical RNA recognition motifs with which it binds target RNAs. Therefore, question arises as to how many of the PND12 cKO testis DEGs resulted because of TDP-43 binding to their RNAs. To this end, we explored the published database of TDP-43 binding RNAs in mouse brain (20, 21). Since TDP-43 is a ubiquitously expressed essential protein, we reasoned that some target RNAs of TDP-43 would be common to both testis and brain. Of the 16,911 genes expressed in our RNA-Seq data, 7055 had a CLIP site (TDP-43 binding site in mRNA). This indicates that TDP-43 target RNAs are shared between brain and testis. Next, to investigate the link between loss of TDP-43 and differential gene expression in testis, we asked how many of the testis DEGs contained TDP-43 CLIP sites. Bioinformatics analysis showed that 825 of 1638 downregulated and 569 of 1036 upregulated DEGs showed overlap with TDP-43 target RNAs (containing a CLIP site) (Fig. 8*D*), which is many more than expected because of chance (one-tailed hypergeometric test *p* value = 4.7e^−14^ and *p* value = 4.2e^−19^, respectively). TDP-43 CLIP-Seq study in mouse brain showed that most downregulated genes after TDP-43 knockdown harbored more TDP-43 CLIP clusters and had exceptionally long introns compared with unaffected or upregulated genes. We compared our testis DEG list with the brain TDP-43 CLIP dataset and found that most upregulated as well as most downregulated genes in cKO testis contain longer introns than nonregulated genes (Fig. 8*E*) (upregulated *versus* nonregulated *p* value = 1.6e^−10^ and downregulated *versus* nonregulated *p* value = 3.2e^−07^) although upregulated genes have slightly longer introns than downregulated genes (*p* = 0.037). For the mean cluster count in introns, upregulated genes have many more clusters than nonregulated genes (*p* = 7.9e^−05^) and a few more clusters than downregulated genes (*p* = 0.024), whereas there was no significant difference in the number of clusters between downregulated and nonregulated genes (*p* = 0.443) (Figs. 8*E* and S6, *C* and *D*). Next, using the brain TDP-43 CLIP-Seq data, we asked what proportion of testis DEGs contains TDP-43 binding sites within the UTRs of target mRNAs. Of the 1036 upregulated genes in the testis, 39 and 144 genes showed overlap with brain RNAs containing TDP-43 binding sites within the 5′ and 3′ UTR, respectively (Fig. S6, *E* and *F*), which is also more than expected because of chance (one-tailed hypergeometric test *p* value = 1.5e^−05^ and *p* = 6.8e^−07^, respectively). Of the 1638 downregulated genes in the testis, only 34 and 153 showed overlap with brain target RNAs within the 5′ and 3′ UTR, respectively, which was not more than expected because of chance (one-tailed hypergeometric test *p* value = 0.25 and *p* value = 0.54, respectively).

### Loss of repressor function of TDP-43

**Figure 9:**
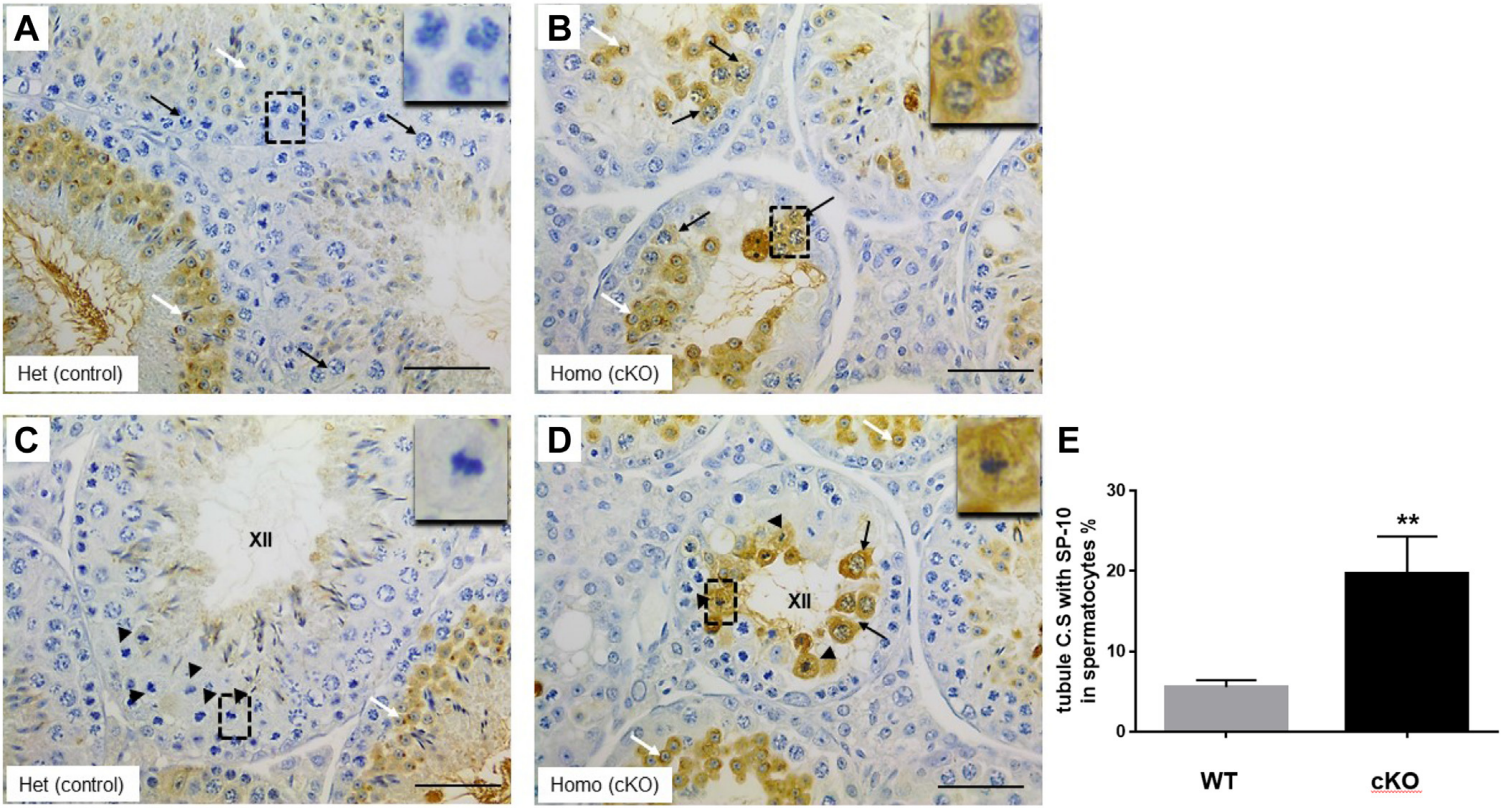
**Premature expression of the Acrv1 gene product SP-10 in spermatocytes of cKO mice.** IHC using antibody to SP-10 on testis cross sections from control (*A* and *C*) and cKO (*B* and *D*) mice. Note that only round spermatids express SP-10 in *A* and *C* (*white arrows*), and the lack of staining in spermatocytes (*black arrows* in *A*). *Insets* in *A* and *C* highlight the lack of SP-10 expression in spermatocytes of control mice. In contrast, several spermatocytes in cKO testis (*B* and *D*) show staining for SP-10 within the cytoplasm (*black arrows* in *B* and *D*). *Arrowheads* in *C* and *D* point to spermatocytes in meiotic division at stage XII. Note SP-10 expression in cKO testis (*arrowheads* in *D*), whereas the equivalent stage XII tubule of the control testis does not show SP-10 expression in spermatocytes (*C*). *Insets* in panels *C* and *D* show this more clearly. The scale bar in *A*–*D* represents 50 μm. *Insets* represent 4.25 (panel *A*) or 5 times (*C* and *D*) magnification of *boxed area* with *dashed lines*. The number of tubule cross sections (C.S) showing SP-10 antibody immunoreactivity in spermatocytes was counted from three cKO and three B6 control mice; at least 60 cross sections were counted per mouse. Percentage of tubule C.S showing SP-10-stained spermatocytes was plotted in panel *E*. cKO, conditional KO; IHC, immunohistochemistry.

Our previous work showed that TDP-43 functions as a transcriptional repressor and that the *Acrv1* gene is a TDP-43 target gene *in vivo* (12, 13). We showed that mutation of TDP-43 binding sites within the promoter of the *Acrv1* gene resulted in premature expression of a reporter gene in spermatocytes of transgenic mice (12). The present cKO mouse model provided an opportunity to ask if the loss of endogenous TDP-43 leads to premature expression of the *Acrv1* gene product (SP-10 protein) in spermatocytes *in vivo*. IHC of testis cross sections showed the typical round spermatid-specific immunoreactivity of the acrosomal protein SP-10 in control mice testis (Fig. 9, *A* and *C*). In contrast, in addition to round spermatid expression, the cKO testes also showed antibody staining within the cytoplasm of spermatocytes (Fig. 9, *B* and *D*). There was a statistically significant increase in the expression of SP-10 within the spermatocytes of the cKO mice (Fig. 9*E*). Thus, lack of endogenous TDP-43 led to premature expression of the *Acrv1* gene in spermatocytes. Taken together, the data indicate that one function of TDP-43 is to repress the transcription of its target genes such as the *Acrv1* gene in spermatocytes.

## Discussion

Infertility affects one in six couples in the reproductive age group in the United States; the male factor accounts for approximately half the number of these cases (22). Compared with the female, male infertility has no treatment options, which is largely because of the idiopathic nature of the disease (23, 24). Therefore, it is important to identify new factors that contribute to male infertility. Our previous study showed aberrant TDP-43 expression in the spermatozoa of infertile men (16). In the present study, conditional deletion of TDP-43 in mouse spermatogonia caused failure of meiotic prophase I leading to maturation arrest and severely reduced fertility. The seminiferous epithelium of adult *Tardbp* cKO mice showed pathology reminiscent of germ cell aplasia seen in human male infertility including meiotic arrest and sertoli cell–only syndrome.

Although TDP-43 is expressed in the intermediate and type B spermatogonia, loss of TDP-43 in these cells did not appear to affect the formation of PL spermatocytes as judged by the expression of the Stra8 protein (Fig. S2, *C* and *D*). Subsequent germ cell types, however, were severely affected. Accumulation of midpachytene stage spermatocytes in cKO mice and relatively low representation of diplotene and diakinesis stages indicate failure to complete prophase I (Fig. 4*A*). Consequently, the cKO mice produced only a low number of sperm, a majority of which were morphologically abnormal. *Tardbp* cKO males were severely subfertile (Fig. 2, *I*–*L*). This shows that TDP-43 plays a critical role in male germ cell differentiation and formation of functional sperm.

### Possible reasons for the failure of meiosis in *Tardbp* cKO testis

In the postnatal testis of mice, RA pathway plays an important role in the commitment of male germ cells to meiosis (25). Expression of Stra8 within the PL spermatocytes is considered as a downstream marker of the RA signaling pathway (26). Gene KO strategy showed that Stra8 is a critical mediator of the RA pathway in the testis (27). Expression of Stra8, however, was not affected in *Tardbp* cKO testes suggesting that TDP-43 might play a role independent of Stra8 during meiosis. Among the genes important for the RA pathway, RBP4, Crbp2, and RAR beta showed decreased gene expression in *Tardbp* cKO testes, whereas Sall4b showed a significantly high level of expression (Fig. 7*A*). Previous studies showed the requirement of RARs and RBP4 for male fertility (19). Thus, reduced expression of RAR, RBP4, and Crbp2 might have impaired proper execution of the RA pathway leading to meiotic failure in *Tardbp* cKO mice. Although PND12 was chosen to capture gene expression changes early in pathogenesis, the observed reduction of gene expression may be due to the loss of meiotic germ cells in the cKO testis (Fig. 6). Future studies will address the mechanisms by which TDP-43 regulates the expression of the above genes.

Loss of TDP-43 resulted in the failure of progression through the pachynema stage of prophase I of meiosis. DSB formation, repair, and homologous recombination are critical events that occur during prophase I. Meiotic chromosome spreads showed that γH2AX signals persisted in pachytene-like spermatocytes in cKO testes. These γH2AX flares/foci on chromosomes indicate delayed DSB repair. Typically, by the time spermatocytes enter late pachytene stage, most of the DSBs should have been repaired, and no γH2AX should have been present on the autosomes. Thus, persistence of γH2AX foci in pachytene-like spermatocytes of cKO mice is indicative of unrepaired DSBs. In support of this idea, qRT–PCR data showed that the expression of both SPO11 (protein generating DSBs) and DMC1 (DSB repair protein) was downregulated in the cKO testes. Extensive γH2AX on meiotic chromosomes could have also resulted from nonhomologous synapsis, resembling the pseudosex body seen in *Spo11* mutant (28). SYCP3 staining showed an abnormal number of chromosomes (Fig. 3, *J*–*M*) in cKO mice. Structure illumination microscopy showed synapsis defects and nonhomologous chromosome pairing and synapsis. Given that DSB formation is critical for homologous searching at leptotene stage and that nonhomologous pairing occurs in *Spo11* mutant mice (28), it is likely that low expression of SPO11 in *Tardbp* cKO mice might have contributed to nonhomologous pairing.

Interestingly, a role for TDP-43 in nonhomologous end joining (NHEJ)–mediated DSB repair has been previously reported. In induced pluripotent stem cell–derived motor neurons, TDP-43 was shown to be recruited at DSB sites, where it interacted with factors of the NHEJ-mediated DSB repair pathway (29). In particular, TDP-43 acted as a scaffold to recruit the break-sealing XRCC4–DNA ligase 4 complex. Depletion of TDP-43 resulted in accumulation of genomic DSBs by impairing NHEJ repair (29). In contrast to mitotically dividing cells, however, DSBs made during meiosis are repaired by homologous recombination (30). Nevertheless, TDP-43 may play an equivalent role in DSB repair during male meiosis by acting as a scaffold to recruit repair proteins, including DMC1 and RAD51. It is interesting to note that similar to the phenotype observed in the present TDP-43 cKO mice, KO of DMC1 or RAD51 in mouse spermatocytes also leads to arrest of meiosis in prophase I (31, 32). Future studies will test if protein complementation using DMC1 and RAD51 would rescue the meiotic arrest caused by TDP-43 cKO.

The asynapsis and nonhomologous synapsis observed in *Tardbp* cKO spermatocytes might have also been caused by abnormalities in the loading of the SC/cohesin proteins. Similarly, loading of cohesions, such as REC8 and RAD21L, may have been affected by the lack of TDP-43. Consistent with this, mRNAs coding for the SC (SYCP 1–3) as well as cohesion proteins (Rec8 and Rad21L) were downregulated in *Tardbp* cKO testes. Previous studies showed that loss of REC8 leads to synapsis between sister chromatids (33). Interestingly, the phenotype of zygotene-like cells of the cKO mice (Fig. 3) resembles that of the *Rec8/RAD21L* mutant mice (34). Loss of TDP-43 also led to reduced expression of a number of other genes important for the progression of meiosis, including Sohlh1, Sohlh2, Hormad1, Hormad2, SC protein 1, Sycp2, and Sycp3 (Fig. 7*B*). KO of all the aforementioned genes disrupted the formation of SC leading to failure of meiosis (35, 36, 37, 38, 39, 40, 41). Decreased expression of meiotic genes in cKO testis raises another possibility that TDP-43 deletion may have affected the expression of a master controller of meiosis such as A-myb (42). Mice lacking A-Myb showed meiotic arrest and failure to complete spermatogenesis (43). Interestingly, A-Myb is differentially spliced in the testis (44), thus leaving it as a potential target for factors such as TDP-43, which regulate alternative splicing.

### Insights gained on the function of TDP-43

It is interesting to note that loss of TDP-43 resulted in high expression of the alternatively spliced Sall4b mRNA in PND12 testis of cKO mice (Fig. 7*A*). Sall4a is the regular spliced product, whereas Sall4b is a product of alternative splicing in which the latter portion of exon 2 gets spliced out (45). Our qRT–PCR primer choice (Table S2) allowed distinction between these two isoforms (19). Several studies showed that Sall4a and Sall4b are expressed in undifferentiated spermatogonia in the mammalian testis, including the mouse (46, 47). Although both isoforms function as transcription factors, Sall4b, which is missing some of the zinc fingers present in Sall4a, binds to a unique set of target gene promoters in embryonic stem cells (45). In the present study, overexpression of Sall4b in PND12 cKO testis suggests that under normal physiological conditions, TDP-43 might be preventing alternative splicing of Sall4 in male germ cells. By scanning the Sall4 gene sequence, we have found two canonical TDP-43 binding sites (TGTGTG) separated by five nucleotides in the +20 position downstream of exon 3 of the mouse Sall4 gene (AY463371 UCSC genome browser). Future experiments will address whether TDP-43 plays a mechanistic role in the skipping of exon 2 splicing of Sall4 mRNA in male germ cells. One previous study showed that deletion of DNMT3L caused elevated Sall4b expression in Thy1+ spermatogonial stem cells, which in turn led to premature stem cell exhaustion and failure of spermatogenesis (48). In the present TDP-43 cKO model, the adult mice showed severe germ cell depletion, which could have resulted from the exhaustion of the spermatogonial stem cell pool over time. It will be of interest to determine if TDP-43 and DNMT3L pathways intersect in the male germ line. Nevertheless, a role for TDP-43 in alternative splicing and exon skipping has been previously established in various mammalian cell types (4, 49, 50). Role for TDP-43 in post-transcriptional gene regulation within the testis is consistent with a similar requirement shown for a number of other RNA-binding proteins, including testis nuclear RNA-binding protein (51), deleted in azoospermia (52), Pumilio RNA-binding protein (53), DEAD-box helicase 5 (54), MRG15 (55), RAN-binding protein 9 (56), and Ptbp1 (57). KO of RNA-binding protein, brunol1, on the other hand, proved to be nonessential for male fertility (58).

Our previous studies demonstrated the repressor function of TDP-43 and its occupancy of the Acrv1 (TDP-43 target gene) promoter in spermatocytes *in vivo* (13). Data from reporter mice bearing TDP-43 binding site mutant Acrv1 promoter suggested that TDP-43 could be involved in preventing the premature expression of the *Acrv1* gene in spermatocytes (12). The present study provided an opportunity to verify if the endogenous TDP-43 represses Acrv1 gene expression *in vivo*. *Tardbp* cKO mice in fact showed premature expression of the Acrv1 gene product (SP-10) within the cytoplasm of the spermatocytes in testis cross sections (Fig. 9). Thus, endogenous TDP-43 appears to be required to prevent premature expression of *Acrv1* gene in spermatocytes *in vivo*. Our earlier study also showed the occupancy of pol II as well as negative elongation factor at the Acrv1 promoter within the spermatocytes suggesting regulation by pausing (13). Premature expression of SP-10 in *Tardbp* cKO spermatocytes seen in the current study lends support to the idea that TDP-43 might play a part in the pausing of Acrv1 gene expression in spermatocytes.

### Transcriptome changes in *Tardbp* cKO testis and bioinformatics analysis

RNA-Seq showed that loss of TDP-43 from male germ cells affects global gene expression; 2674 genes were differentially expressed in the *Tardbp* cKO testis. Loss of TDP-43 caused transcript levels to change in both directions although the trend was that there were more downregulated than upregulated DEGs (Figs. 8*A* and S5*A*). TDP-43 is known to play multiple roles in gene regulation including repression of transcription, alternative splicing, and mRNA stability. Improper alternative splicing and loss of mRNA stability could have led to transcript decay and downregulation of gene expression, whereas loss of transcriptional repressor function might have elevated the transcript levels of target DEGs. KEGG pathway analysis showed RNA transport, cell cycle, DNA replication, and basal transcription factor pathways among the top affected pathways. This explains, at least in part, the phenotype seen in *Tardbp* cKO testis and sperm. The differentiating germ cells have intercellular bridges through which RNAs are transported, and KOs affecting this transport have been shown to be detrimental to spermatogenesis (59, 60). Similarly, the failure to progress through meiosis in *Tardbp* cKO testis is consistent with the derailment of cell cycle and DNA replication pathways, which are critical for spermatocytes to complete meiosis properly. GO analysis showed that several BPs associated with spermatogenesis are affected, including meiotic cell cycle, synapsis, homologous chromosome segregation, and homologous recombination. The aforementioned observations suggest that TDP-43 is critical for the proper execution of meiotic prophase I and subsequent germ cell differentiation.

Finally, bioinformatics comparison of RNA-Seq data with previously published mouse brain TDP-43 CLIP-Seq studies provided a plausible link between the RNA-binding function of TDP-43 and differential gene expression in *Tardbp* cKO testis and revealed interesting insights. First, 825 of the 1638 downregulated and 569 of 1036 upregulated DEGs contained one or more CLIP sites. Thus, approximately 52% of the DEGs in *Tardbp* cKO testis contained TDP-43 binding sites in their RNAs, which increases the likelihood that they are direct targets of TDP-43. The remaining 48% DEGs likely include testis-specific genes (hence not found in the brain CLIP-Seq database), genes regulated by the transcriptional function (promoter binding but not RNA binding), or those that are indirectly affected by the loss of TDP-43. Second, many of the testis DEGs contained exceptionally long introns and higher mean TDP-43 binding cluster count as reported for the TDP-43 regulated genes in mouse brain (20, 21). The difference, however, is that both upregulated and downregulated DEGs in testis showed the aforementioned trend, whereas only downregulated genes in the brain showed longer introns. This is interesting and signifies tissue-specific differences in the function of TDP-43. Third, only a minor portion of the upregulated (none of the downregulated) testis DEGs contained TDP-43 CLIPs in the UTRs of RNA, indicating that at the RNA level, TDP-43 is more likely to function as a splicing regulator than in maintaining steady-state levels in the testis.

In summary, the present study shows that the ubiquitously expressed and evolutionarily conserved TDP-43 is essential for spermatogenesis and male fertility. This is a significant finding given the rise in incidence of infertility in men and the need to know the causative factors. Our work also highlights that loss of function of TDP-43 itself leads to pathology in the testis. This has larger implications for other TDP-43 proteinopathies. As a central pathological hallmark, nuclear clearance of TDP-43 accompanied by its cytoplasmic aggregates in neurons and glia has been documented in neurodegenerative diseases. Opinions differ on whether toxic gain of function of cytoplasmic aggregates or loss of its normal function in the nucleus leads to neuronal loss (61). Drug development efforts have focused primarily on countering the toxic gain-of-function aspects of TDP-43 pathology (62). The present study warrants that equal emphasis be placed on ameliorating nuclear functions resulting from loss of TDP-43 from the nuclei of motor neurons and glial cells of patients with neurodegenerative diseases.

## Experimental procedures

### Generation of *Tardbp* cKO mice

*Tardbp* cKO mice in which exon 3 of the *Tardbp* gene is flanked by *loxP* sites were previously reported (10). For male germ cell–specific deletion, we used the Stra8–iCre deleter mouse strain purchased from the Jackson Laboratory (STOCK Tg(Stra8-icre)1Reb/J; stock no: 008208). DNA extracted from tail biopsies was used for genotyping as described previously (10). The sequence of the primers will be made available upon request.

### Antibodies used in the study

Guinea pig anti-TDP-43 polyclonal antibody was reported previously (15). Antibody to Stra8 was a kind gift from Dr Michael Griswold, Washington State University. Anti-SYCP3 (Abcam; 15093), anti-γH2AX antibody (Millipore Sigma; 05-636), and anti-Sox9 antibody (Millipore; AB5535) were obtained from commercial sources. Anti-γH2AX antibody used for IHC was a mouse monoclonal from Millipore 05-636. Guinea pig anti-SP-10 polyclonal antibody was reported previously (63).

### Testis and sperm analysis

Mice were euthanized following the procedures approved by the Institutional Animal Care and Use Committee of University of Illinois Urbana Champaign. Testes were weighed prior to the transfer of one testis to Bouin's fixative solution and the other to the −80 °C freezer. Cauda epididymidis were punctured to release sperm into PBS at 37 °C for 20 min. Sperm counting was done using a hemocytometer. Sperms were stained with hematoxylin, and morphological parameters were observed under the light microscope, and images were captured. Histopathology of the testis was evaluated by H&E staining. The diameter of the seminiferous tubules cross sections from homozygous cKO mice and littermate controls was compared by measuring the shortest diameter distance in at least 30 tubule cross sections at different depths per biological replicate of each genotype using ImageJ software (the National Institutes of Health).

### IHC

Bouin's fixed testes were processed for paraffin embedding using a Tissue-Tek VIP 1000 processor (Sakura Finetek). About 4-micron thick sections were cut using the Leica RM2125 RTS rotary microtome (Leica Biosystems) and mounted on glass slides. Slides were deparaffinized with xylene and hydrated through a series of graded ethyl alcohols. Antigen retrieval was performed using citrate buffer (pH 6.0) in a vegetable steamer for 60 min. Endogenous peroxidase was blocked using 3.0% hydrogen peroxide for 10 min. Background Punisher (Biocare Medical) was used for 20 min to block nonspecific background (BG). The sections were incubated for 1 h at room temperature in the primary antibody. Following rinsing, the sections were incubated with secondary antibodies. 3,3′Diamenobenzidine (Innovex Biosciences, Inc) was used as the chromogen with an incubation time of 5 min. Slides were counterstained with hematoxylin, dehydrated, cleared, and mounted.

### Apoptosis

Mouse testis cross sections were stained for the detection of apoptosis using ApopTag Peroxidase *In Situ* Apoptosis Detection Kit (Sigma–Aldrich; S7100) per manufacturer's instructions. Three cKO and three B6 WT control males were used. About 50 cross sections per animal were counted; cross sections containing three or more apoptotic cells were counted as positive.

### Sperm analysis

For sperm count and morphology analysis, caudal epididymidis of adult cKO and C57bl/6 controls was collected, minced, and incubated in 1 ml of PBS at 37 °C for 20 min to release spermatozoa. Sperms were counted using a hemocytometer under an optical microscope (Laxco). For morphology analysis, sperms were diluted in PBS, and 30 μl drops were placed on slides. The samples were air dried, incubated with 4% paraformaldehyde in PBS for 10 min at RT, washed three times with PBS, and then incubated in hematoxylin (Ricca) for 2 min. Slides were washed with water and mounted in Entellan (Millipore). The morphology of at least 160 random spermatozoa per sample was analyzed under 100× magnification and classified as normal (no visible alterations), detached head, deformed head, or bent/curved tail.

### Statistical tests for histological and morphological analysis

Statistical significance was evaluated by Student's *t* test using Prism software (GraphPad Software, Inc) when appropriate. All experiments were performed with at least three biological replicates. Data are described as mean ± SD, with differences considered significant when *p* < 0.05.

### Flow cytometry

Testicular germ cells were stained with a nuclear stain and subjected to flow cytometry for classification based on DNA content, as described previously (64). Briefly, testes were decapsulated, tubules were minced, and washed two times with Dulbecco's modified Eagle's medium (Thermo Fisher Scientific) to eliminate interstitial cells. Tubule pieces were incubated for 20 min at 34.5 °C with collagenase type IV (2 mg/ml; Sigma) and DNAse I (40 μg/ml; Sigma). Then, the tubules were washed two times in Dulbecco's modified Eagle's medium and resuspended in trypsin (10 mg/ml), collagenase IV (1 mg/ml), hyaluronidase (1.5 mg/ml), and DNase I (40 μg/ml) for 15 min at 34.5 °C. The cells were passed through a 45-mm nylon mesh, centrifuged at 300*g* for 5 min, and suspended in PBS. Finally, cells were fixed in 4% paraformaldehyde (PFA), washed with PBS, stained with Hoechst (1:500 dilution; Thermo Fisher Scientific), and analyzed using a flow cytometer (Cytek Aurora).

### Fertility trials

Four adult F/-, iCre+ males (cKO) and five adult C57B6 males (control) were used in fertility trials. In each case, the male was paired with a C57B6 female, and the breeding cages were maintained for 6 months. The females were routinely checked for plugs. The number of litters produced and pups born to the cKO and control group was recorded for the 6 months' duration.

### Chromosomal spreading of spermatocytes

Chromosome “spreads” were prepared from mouse testes according to previous publication (65, 66). After euthanizing the mice, testes were dissected out and punctured to isolate the seminiferous tubules and immersed in PBS. One-fourth of the tubules was incubated in hypotonic extraction buffer (pH 8.2) (30 mM Tris [pH 8.0], 50 mM sucrose, 17 mM citric acid, 5 mM EDTA, 0.5 mM DTT, and 1 mM PMSF) and placed on ice for 20 min. A small section of the seminiferous tubules transferred from hypotonic extraction buffer to 35 μl of 0.1 M sucrose solution and minced by a scalpel. Tubules were minced until a cloudy cell suspension is formed, and an additional 65 μl of sucrose was added into the suspension. Glass slides were coated in 100 μl 1% PFA containing 0.1% Triton X-100. To each slide, 18 μl of tubule sucrose suspension was slowly added to the PFA solution and evenly spread. Slides were left in a sealed humidity chamber overnight. The next morning, the lid was slightly opened for 30 min and then fully removed to allow slides to dry. The slides were placed in a Coplin jar filled with distilled water and washed on a shaker for 5 min at room temperature. Following two more washes in 0.4% Photo-Flo 200 (Kodak; 1464510) solution, slides were taken out to air dry. Slides were then prepared for immunostaining or stored at −80 °C.

### Immunostaining of chromosomal spreads

Slides containing spermatocyte chromosomal spreads from adult (3 months old) TDP-43 cKO and WT mice were stained with 4′,6-diamidino-2-phenylindole (*blue*) and antibodies to TDP-43 (12), anti-SYCP3 (Abcam; 15093), and anti-γH2AX antibody (Millipore Sigma; 05-636). Chromosome spreads were washed twice with Tris-buffered saline with 0.1% Tween-20 (TBST). Next, the slides were incubated with 500 μl of 10% antibody dilution buffer (ADB) blocking solution (0.3% bovine serum albumin, 10% normal goat serum, and 0.005% Triton-X-100 in TBS) twice for 15 min at room temperature. Primary antibodies were diluted in ADB. Each slide was incubated with 100 μl of primary antibody (anti-TDP-43 diluted at 1:500; anti-SYCP3 diluted at 1:200; and anti-γH2AX diluted at 1:500) and covered with a plastic cover slip overnight in a wet dark box at room temperature. Plastic coverslips were removed by washing with TBST and carefully peeled with tweezers. Once again, slides were incubated in 10% ADB twice for 15 min. The goat secondary antibodies (antimouse 594 [Molecular Probes; A11020; 1:1000 dilution]; anti-rabbit 488 [Molecular Probes; A11070; 1:1000 dilution]; anti-Guinea Pig [Life Technologies; A21435; 1:1000 dilution]) were diluted in ADB and added to slides. Slides were covered with a plastic cover slip and incubated at 37 °C in a dark wet box for 1 h. Slides were then removed from the incubator and allowed to equilibrate to room temperature. Coverslips were removed as aforementioned, and slides were washed three times with TBST. After TBST washes, the slides were then washed two more times with distilled water and left to air dry for 5 min. Finally, slides were mounted with 25 μl of Prolong mounting media (Fisher Scientific; P36970) and covered with a glass coverslip.

### Imaging and data analysis

Using Nikon A1R confocal microscopy, individual spermatocytes were captured and staged into the following seven stages: zygotene, early pachytene, midpachytene, late pachytene, early diplotene, late diplotene, and diakinesis. Assignment of the stages was based on SYCP3 staining patterns.

With the NIS-Elements imaging software (Nikon), an individual region of interest (ROI) was drawn around each nucleus. Each ROI was duplicated in a neighbor region where there is no DNA staining. The software calculated the sum intensity of TDP-43, SYCP3, and 4′,6-diamidino-2-phenylindole. The duplicated ROI was used to account for BG intensity and subtracted out of the sample's sum intensity. The following calculation was used to determine the ratio between TDP-43 and SYCP3:

$$\text{TDP}-43\;\text{and}\;\text{SYCP}3\text{:}\frac{\text{Sum}\;\text{of}\;\text{TDP}43\;\text{intensity}-\text{Sum}\;\text{of}\;\text{TDP}43\;\text{BG}\;\text{intensity}}{\text{Sum}\;\text{of}\;\text{SYCP}3\;\text{intensity}-\text{Sum}\;\text{of}\;\text{SYCP}3\;\text{BG}\;\text{intensity}}$$

### RNA extraction and cDNA synthesis

Testis samples were prepared using a handheld homogenizer for 1 min. The homogenizer was cleaned between each sample using four alternating washes of 100% ethanol and sterile deionized water for at least 1 min in each. RNA was extracted from the lysate using spin columns in the PureLink RNA Mini Kit (Invitrogen; catalog no.: 12183018A). Samples were stored at −80 °C until the RNA (1 μg) was reverse transcribed using the Qiagen Quantitect RT Kit (Qiagen, Inc). The cDNA samples were stored at −20 °C until further analysis.

### Primer design for qRT–PCR

Oligonucleotide primers (Tables S1 and S2) were designed using the National Center for Biotechnology Information (NCBI) Primer Design Tool, seeking to span an exon–exon junction with an intervening intron where possible. All targets were tested prior to use on “no reverse-transcriptase” RNA samples to check for genomic DNA contamination and the amplification of nonspecific targets. Acceptable primers had no amplification, or the Ct value of the no reverse transcriptase sample was at least four cycles greater than target cDNA amplification, and the melting temperature was a single and clear peak.

### qRT–PCR analysis

qPCR was performed on a QuantStudio 3 Thermocycler (Thermo Fisher Scientific) using SybrGreen PowerUp Master Mix (Thermo Fisher Scientific). The 20 μl reaction included 1 μl target cDNA template, 10 μl SybrGreen Master Mix, 8 μl nuclease-free water, 0.5 μl forward primer, and 0.5 μl reverse primer. The reactions were incubated in a 96-well plate at 95 °C for 2 min, followed by 40 cycles of 95 °C for 30 s, 60 °C for 30 s, and 72 °C for 30 s. All qPCR plates included a negative control, actin as the reference gene for each sample, and all reactions were performed in triplicate. The cycle threshold (Ct) is the number of cycles required for the fluorescent signal to exceed BG levels. Relative gene transcription changes were calculated using the 2^−ΔΔCt^ method (67):

ΔCt(sample)=Ct(targetcDNAsample)−Ct(referencecDNAsample)

ΔCt(control)=Ct(targetcDNAcontrol)−Ct(referencecDNAcontrol)

ΔΔCt=ΔCt(sample)−ΔCt(control)

$$\text{Normalized}\;\text{target}\;\text{gene}\;\text{expression}\;{\text{level}=2}^{-\Delta \Delta \text{C}}$$

### Statistical analysis of qRT–PCR data

All results are the averages of three independent *Tardbp* cKO mice samples compared with controls. Data are expressed as the mean ± SEM for a 95% confidence interval. Statistical comparisons of ΔΔCt were made by Student's *t* test. Statistically significant difference was indicated with *p* < 0.05.

#### RNA-Seq and pathway analysis

Total RNA was isolated from the whole testes of PND12 *Tardbp* cKO (n = 3) and WT control (n = 3) mice and sent to the Roy J. Carver Biotechnology Center at the University of Illinois Urbana-Champaign for preparation, sequencing, and analysis. The RNA-Seq libraries were prepared with the TruSeq Stranded mRNAseq Sample Prep kit (Illumina), then pooled, quantitated by qPCR, and sequenced on one S4 lane for 101 cycles from one end of the fragment on a NovaSeq 6000. Fastq files were generated and demultiplexed with the bcl2fastq, v2.20, Conversion Software (Illumina), and adaptors were trimmed. Salmon (version 1.2.0) (68) was used to index the NCBI *Mus musculus* Annotation Release 109 transcriptome using the decoy-aware method with the entire GRCm39 genome as the decoy sequence. Then quasi-mapping was performed to map reads to the transcriptome with additional arguments --seqBias, --gcBias, --numBootstraps=30, --validateMappings, and --recoverOrphans to help improve the accuracy of mappings.

The remaining statistical analyses were done in R, version 4.0.4 (https://www.R-project.org/) using packages as indicated later. Gene-level counts were estimated from transcript-level counts using the “bias corrected counts without an offset” method from the tximport package (version 1.18.0). This method provides more accurate gene-level count estimates and keeps multimapped reads in the analysis compared with traditional alignment-based method (69). Genes without at least 0.5 counts per million in at least three samples were considered “not expressed” and filtered out. The trimmed mean of means (70)–normalized log2 count per million values were calculated and tested for differential expression using the limma-trend method in the limma package (71) (version 3.48.0) using a final model that included a processing batch effect (72). Multiple test correction was done using the FDR method (73), and FDR *p* value < 0.05 was used as the threshold for significance. Over-representation testing of KEGG pathways (updated on April 1, 2021) was done using a one-tailed hypergeometric test separately for upregulated and downregulated gene sets using the EGSEA (74) package (version 1.18.1).

GO enrichment analysis was done using the Bioconductor package EdgeR (75). GO database for the analysis was extracted from a genome-wide annotation for mouse (org.Mm.eg.db). For GO term types, BP and MF, the top 15 GO terms were selected for making the graph based on −log10(*p* value) with the criteria of *p* value less than 10^−5^. For comparing germ cell development–related GO, GO terms were selected from Mouse Genome Informatics GO Browser and categorized into three types manually: spermatogenesis, spermatogonia development, and spermiogenesis.

### Comparison of RNA-Seq data with previously published TDP-43 CLIP-Seq data

We compared the lists of upregulated and downregulated genes with genes identified to have a TDP-43 binding site using CLIP-Seq within or nearby (“CLIP sites”) (21). Of the 16,911 genes expressed in our RNA-Seq data, 7055 had a CLIP site. We tested whether more of the upregulated and downregulated genes than expected had a CLIP site using a one-tailed hypergeometric test from the VennDiagram package, version 1.6.20 (https://CRAN.R-project.org/package=VennDiagram). We also performed the same test of over-representation of genes with CLIP sites specifically in their 3′ or 5′ UTR. To see if intron length or number of CLIP sites in or near a gene is related to a gene's expression after cKO of *Tardbp*, we followed the method of Lagier–Tourenne *et al.* (21) and ranked the genes with at least one CLIP cluster based on descending order of fold change, then compared the most upregulated genes (0–5 percentile), nonregulated genes (52.5–47.5 percentile), and downregulated genes (95–100 percentile); because the standard deviations between the groups were very different, we used nonparametric pairwise Wilcoxon rank sum tests with Bonferroni-corrected *p* values to test for differences in intron length and number of CLIP clusters.

## Data availability

The RNA-Seq data (https://www.ncbi.nlm.nih.gov/geo/info/linking.html) reported in this publication have been deposited in the Gene Expression Omnibus of NCBI and are accessible through GEO Series accession number GSE17573 (https://www.ncbi.nlm.nih.gov/geo/query/acc.cgi?acc=GSE17573). All the RNA-Seq data contained within the article are located as aforementioned (GSE17573). Histopathology of testes of 30 separate cKO mice as well as histopathology of the PND15 cKO mice were reported as “data not shown” in the article. These data will be shared upon reasonable request by the corresponding author (preddi@illinois.edu).

## Supporting information

This article contains supporting information (19).

## Conflict of interest

The authors declare that they have no conflicts of interest with the contents of this article.
