# Supplementary material for: Loss of TDP-43 in male germ cells causes meiotic failure and impairs fertility in mice
Source: J Biol Chem. 2021 Sep 29;297(5):101231. doi: 10.1016/j.jbc.2021.101231 (PMC8569592; doi:10.1016/j.jbc.2021.101231)
Supplement: Supplemental Figures S1–S6 [file mmc1.pdf]

Supporting Information

Loss of TDP-43 in male germ cells causes meiotic failure and impairs fertility in mice

Kaitlyn M. Campbell*, Yiding Xu*, Chintan Patel*, Jeremy M. Rayl, Helena D. Zomer, Hari Prasad Osuru, Michael Pratt, Patcharin Pramoonjago, Madeline Timken, Lyndzi M. Miller, Abigail Ralph, Kathryn M. Storey, Yiheng Peng, Jenny Drnevich, Clotilde Lagier-Tourenne, Philip C. Wong, Huanyu Qiao, Prabhakara P. Reddi

Supplemental Figure 1

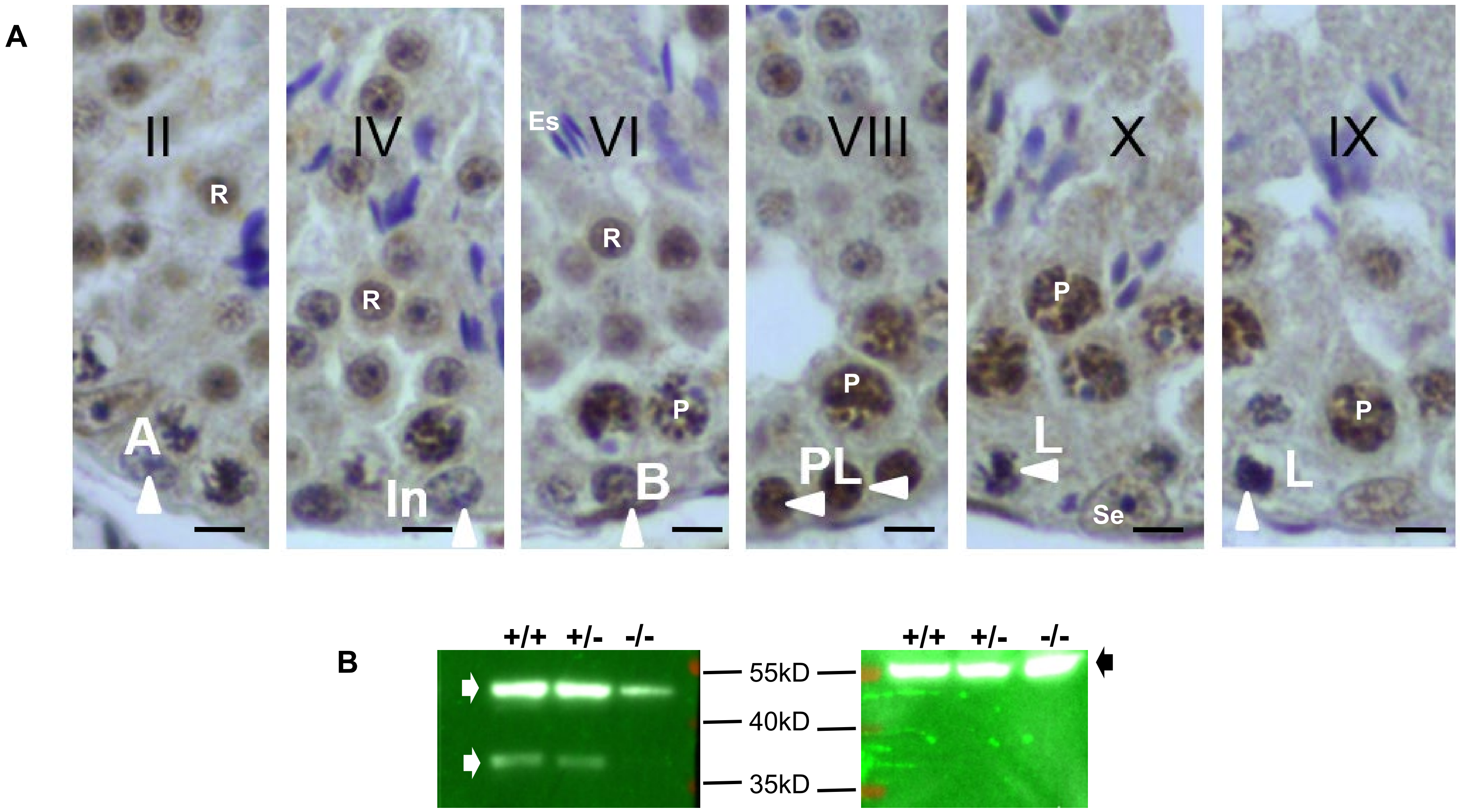

Ab: TDP-43 Alpha tubulin

Supplemental Figure 1: (A) Immunohistochemistry of mouse testis cross sections showing TDP-43 expression in various cell types of the testis. Note that TDP-43 is highly expressed in the nuclei of preleptotene (PL) and pachytene (P) spermatocytes. Roman numerals indicate the stages of the cycle of seminiferous epithelium. Type A (A), Type B (B), and Intermediate spermatogonia (In); Preleptotene (PL), Leptotene (L), and pachytene (P) spermatocytes; Round (R), and Elongated (Es) spermatids, and Sertoli cells (Se) are depicted. Scale bar = 15 μm. Panel B: Twenty micrograms each of testis extracts from WT (+/+), heterozygous (+/-), and homozygous (-/-) Tardbp cKO mice were run by SDS-PAGE in duplicate and immunoblotted with TDP-43 and alpha tubulin antibodies separately. The white arrow heads indicate the 43 kD and 35 kD TDP-43 bands; black arrow head shows the 55kD alpha tubulin band. Note that there is no difference in TDP-43 levels between the +/+ and +/- testis, indicating compensation. In the -/- testis TDP-43 is reduced but not absent because of contribution from the somatic cells of the testis.

Supplemental Figure 2

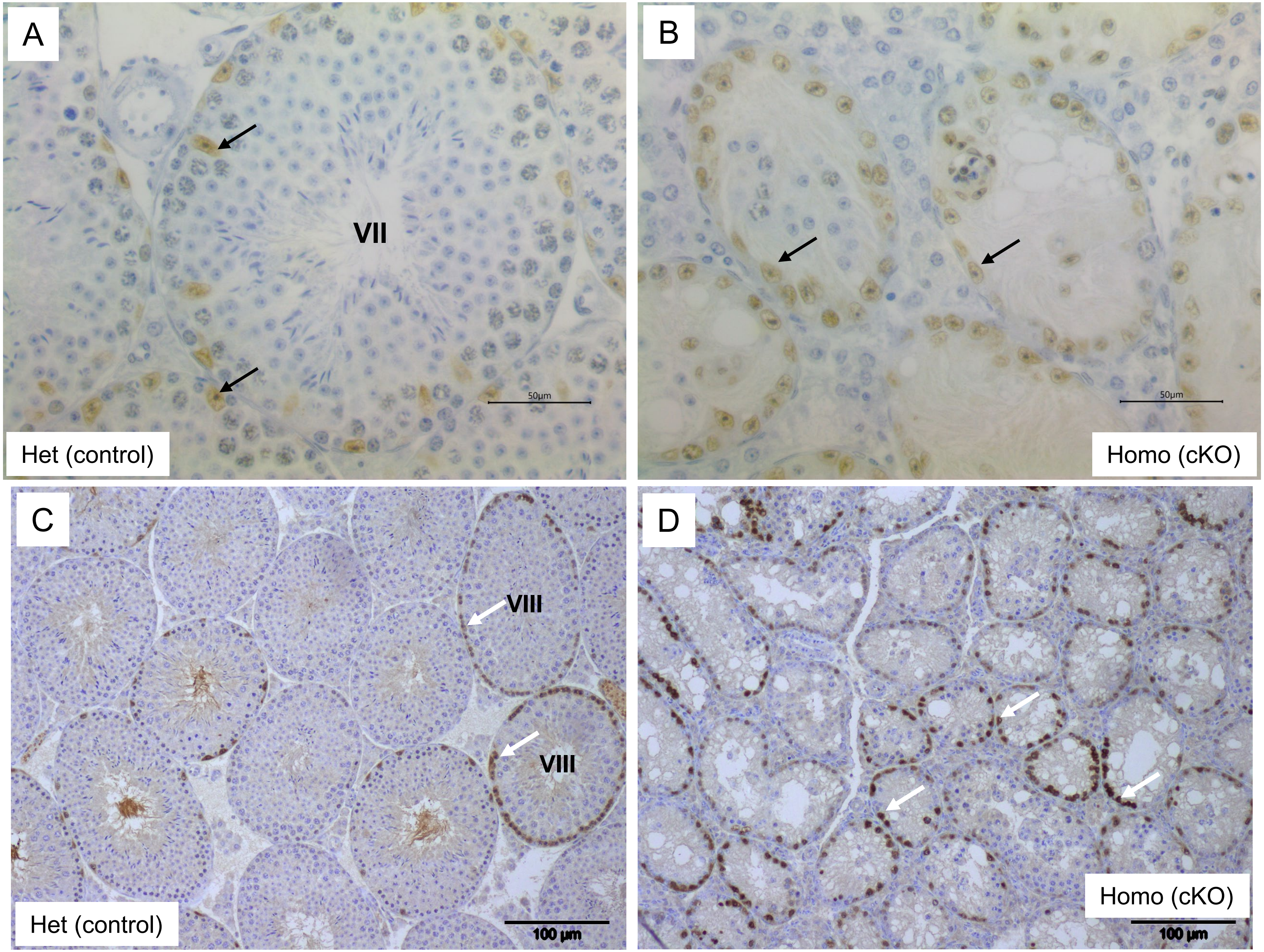

Supplemental Figure 2: A-B: IHC with Sox 9 antibody shows that only Sertoli cells (black arrows) remain in the seminiferous epithelium of a 7 month old cKO mouse testis (B). Control testis shows a full contingent of germ cells but only Sertoli cells are marked by Sox 9 (A). IHC with Stra8 antibody of PND35 mice (white arrows) shows that preleptotene spermatocytes of control mice express Stra8 at stage VIII indicating commitment to meiosis (C). cKO testis also show preleptotene spermatocytes expressing Stra8 although the stages are not easily discernible (D). Scale bar in A-B = 50 μm and C-D = 100 μm.

Supplemental Figure 3

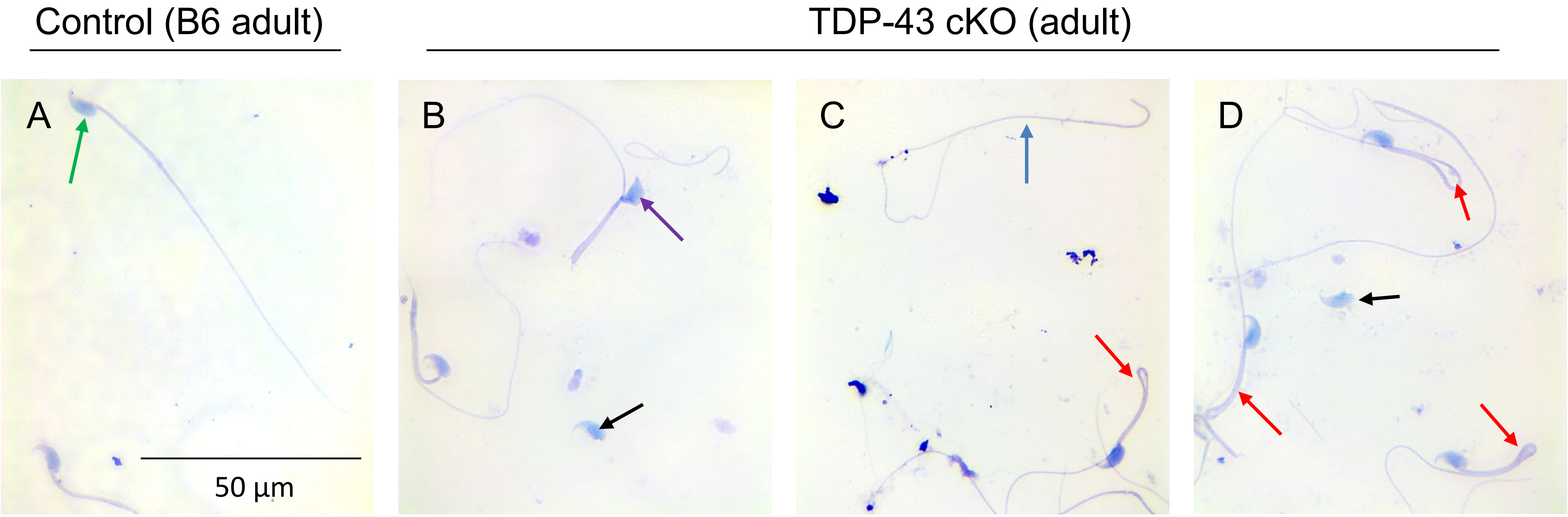

E	Animal	Normal	Deformed Head	Detached Head	Curved/Bent tail
	B6 Average (n=5)	78.4 ± 3.0%	8.5 ± 2.0%	5.3 ± 3.4%	3.8 ± 2.0%
	cKO Average (n=2)	5.7 ± 2.1%	21.3 ± 2.1%	28.25 ± 6.0%	55.0 ± 18.3%

Supplemental Figure 3: Sperm deformities observed in TDP-43 cKO mice (A-D) and quantitation (E). Wild type sperm (A, green arrow) show normal morphology of mouse sperm head while spermatozoa from the cKO mice show deformed head (purple arrow in B), head-less sperm (blue arrow in C), detached head (black arrow in D), and sperm in which the head and the mid-piece are bent over the principal piece of the sperm tail (red arrows in C and D). Table in panel E shows quantification of the data.

Supplemental Figure 4

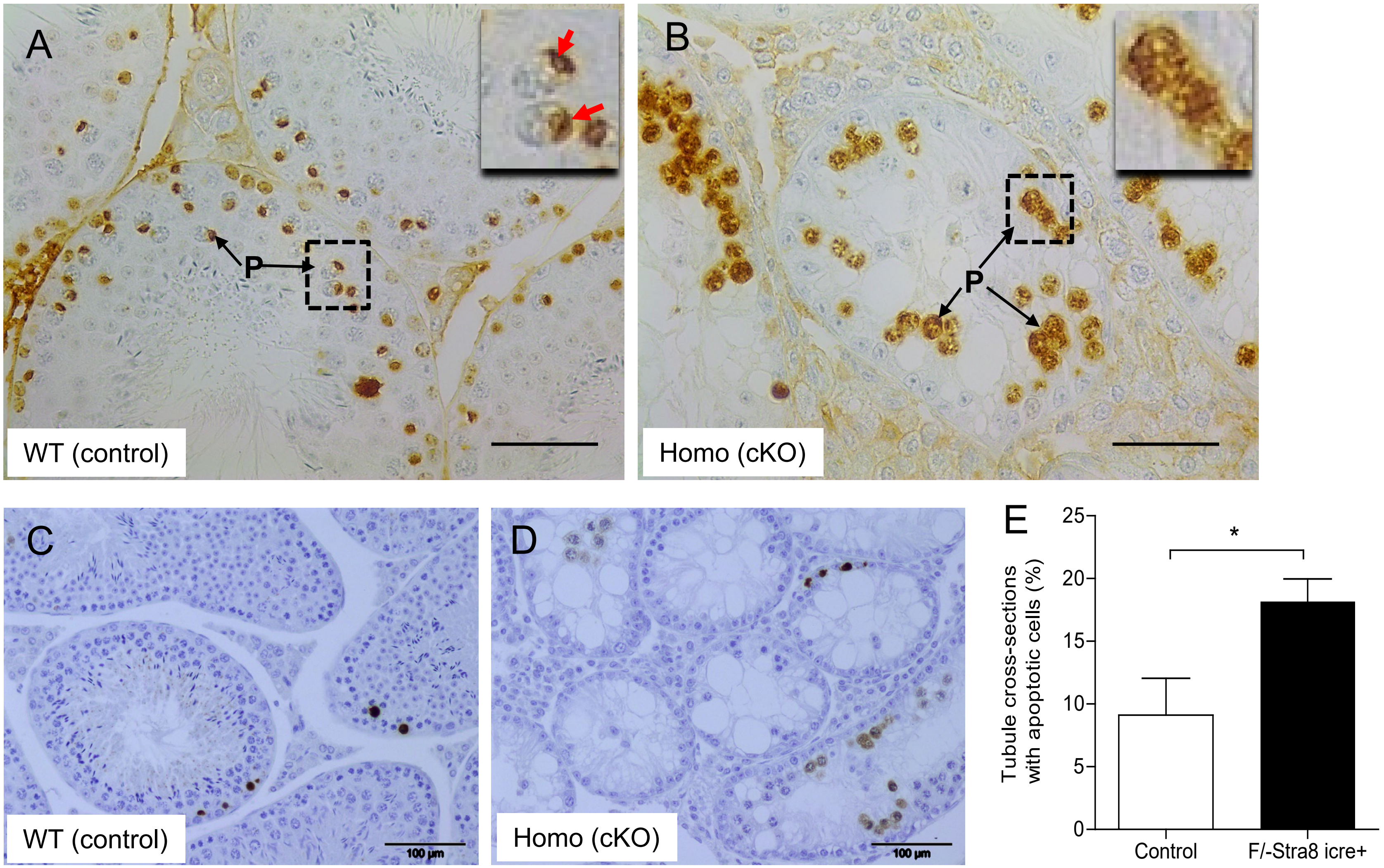

Supplemental Figure 4: IHC with γH2AX antibody showing staining restricted to XY body in pachytene spermatocytes in adult WT control mouse testis (A) whereas in age-matched cKO mice the γH2AX staining is spread out in the entire nuclear region of all of the meiotic cells (B) supporting the IF data shown in Figure 4 B-E. Note that inset in A shows the XY body (red arrows) whereas the inset in B shows staining all over the nucleus. Insets represent 4.5 time magnification of boxed areas outlined by dashed lines in A and B. TUNEL staining showing apoptotic cells within the seminiferous tubules of adult WT control (C) or cKO mice (D). Scale bar in A-B = 50 µm and C-D = 100 µm. Quantification of the percentage of tubule cross sections showing 3 or more apoptotic cells (E).

Supplemental Figure 5

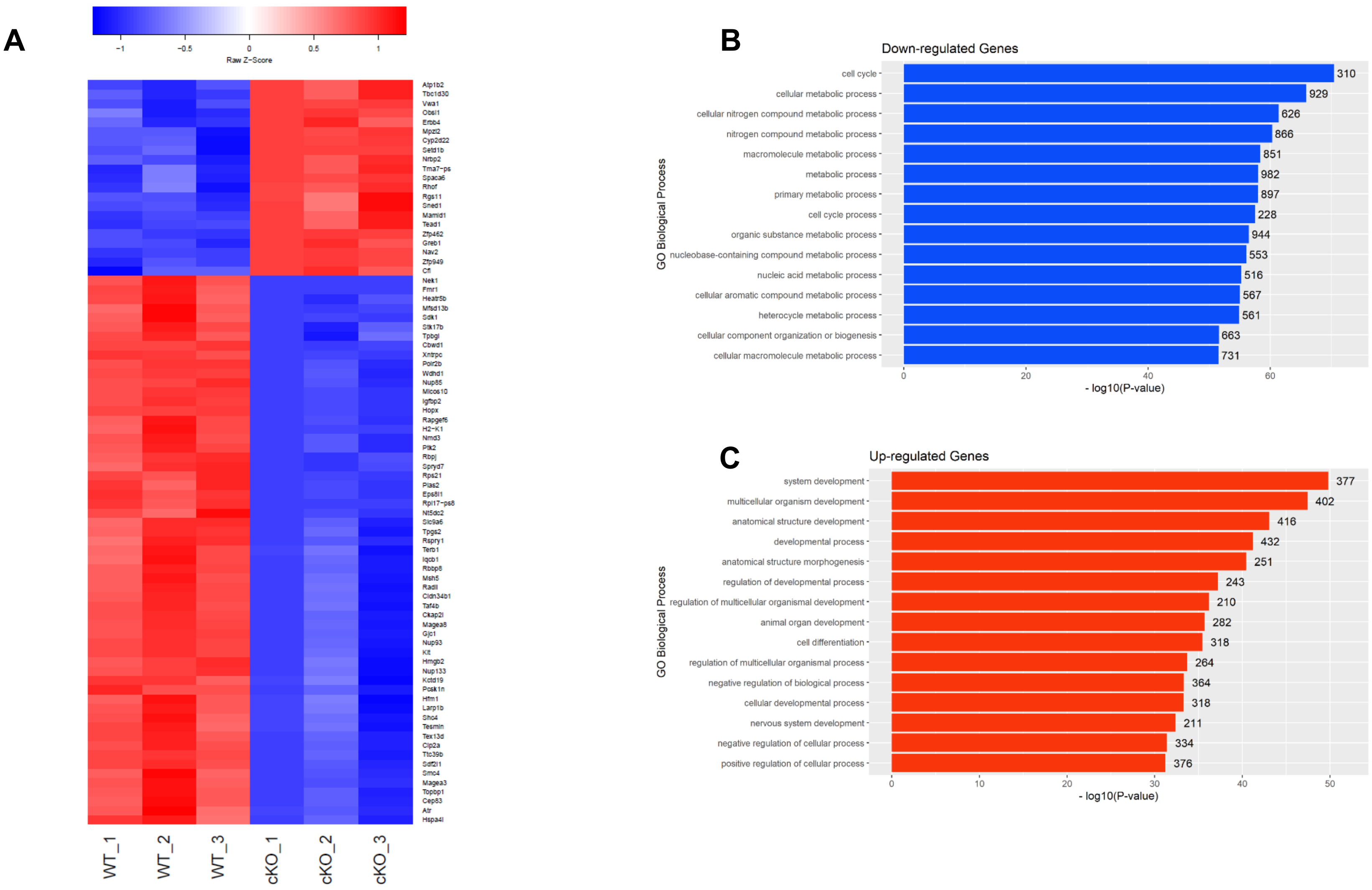

Supplemental Figure 5: (A) Heatmap showing the top 80 differentially expressed genes (DEG) in Tardbp cKO testis; 21 upregulated and 59 downregulated DEGs. (B-C) Top-enriched GO biological processes for upregulated (FDR < 0.05) and downregulated genes (FDR < 0.05), respectively, in Tardbp cKO testis; parenthesis shows the number of DEGs in each biological process.

Supplemental Figure 6

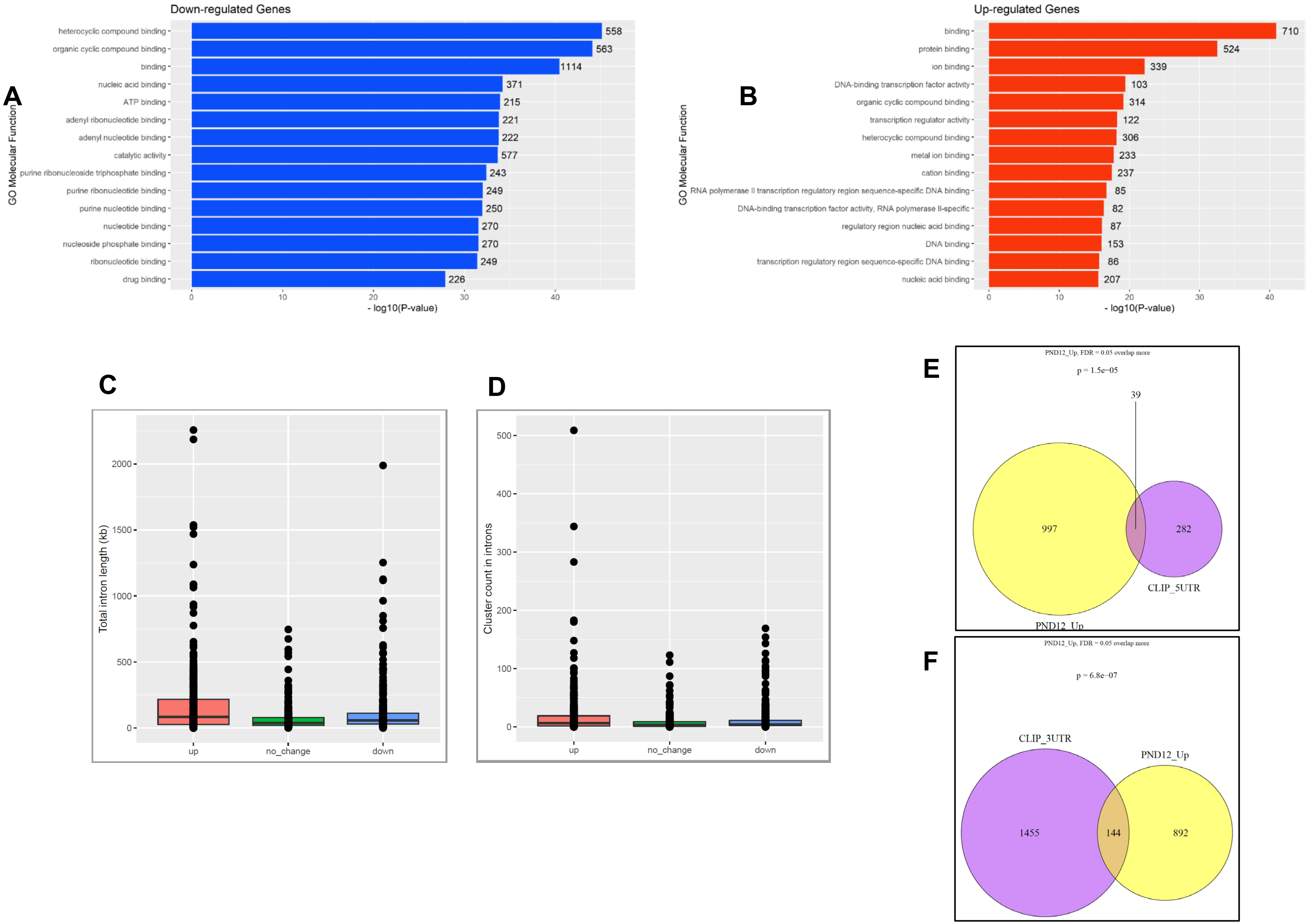

Supplemental Figure 6: (A-B) Top-enriched GO molecular functions for upregulated (FDR < 0.05) and downregulated genes (FDR < 0.05), respectively, in Tardbp cKO testis; parenthesis show number of DEGs in each molecular function. (C-D) Box plots of the 354 most up-regulated, middleest 354 non-regulated, and 354 most down-regulated (354 = 5% of genes) showing the DEGs contain longer introns compared to non-regulated genes (C), and that up-regulated genes have many more clusters compared to non-regulated and down-regulated genes (D). (E-F) Venn diagram showing comparison of upregulated testis DEGs with brain-expressed genes identified to have TDP-43 binding sites (CLIP) in their 5' or 3' UTRs using a hypergeometric test from the VennDiagram package v1.6.20. See text for statistical values for C-F.
