# Supplementary material for: Loss of TDP-43 in male germ cells causes meiotic failure and impairs fertility in mice
Source: J Biol Chem. 2021 Sep 29;297(5):101231. doi: 10.1016/j.jbc.2021.101231 (PMC8569592; doi:10.1016/j.jbc.2021.101231)
Supplement: Supplemental Tables S1 and S2 [file mmc2.pdf]

Table S1. Primers used for quantitative RT-qPCR for meiotic gene targets.

Gene	Accession number	Primers	Position (nucleotide)	Size (nucleotide)
Dmc1	NM_010059.3	5'-GGTGGCATTGAAAGTATGGCAA-3'	521-619	99
		5'-TGGAAGTTGAGCTGTCACACA-3'		
Hormad1	NM_001289534.1	5'-TTCCCTCAGGCGGTGC-3'	36-118	83
		5'-CAATGCACTCAGGGAAGCTG-3'		
Hormad2	NM_029458.1	5'-ACACAAATCCCAAGGAACCTGA-3'	563-665	103
		5'-CTTGTGCTGCTGCTGTCAAA-3'		
SCC3	NM_009282.4	5'-CCAGTGTTCAGGATGTCGAG-3'	797-906	110
		5'-AGTCACCACTGTCCTCATCAAAT-3'		
Spo11	NM_012046.2	5'-TAGTGCCACGGCTACTGCT-3'	640-748	109
		5'-GAGCCGCTGAAATGTTGCAT-3'		
Mei1	NM_028897.3	5'-CAGGACCAGAGAGTCTGCATC-3'	270-379	110
		5'-GTGAGCTGGACAAGGACTTCA-3'		
Msh4	NM_031870.3	5'-CCAGGCCGAGTACGGAAGG-3'	390-485	96
		5'-TGTTTCCAAATGATGAACCTGGC-3'		
Prdm9	NM_144809.3	5'-AGAGTGGACACCTAAGCAACA-3'	321-410	90
		5'-GGCATTCTAGATGATTCCTTCTGC-3'		
Rad21L	NM_001276400.1	5'-TGAATTGGAGTCATCAGGCAG-3'	1386-1495	110
		5'-ACTGCATTCCCATTTTGTTGAA-3'		
Rec8	NM_020002.3	5'-TTTCAACAGTGCCAGTACCTT-3'	489-598	110
		5'-AAGCTAGGTAGGTCAGCCTC-3'		
Sycp1	NM_011516.2	5'-GCGGGAGGAGACTCCAACTA-3'	211-317	107
		5'-GCAGGATCTTTATCAATGTTTTCCC-3'		
Sycp2	NM_177191.3	5'-GCCCACAGCCTAAAGTGTCT-3'	89-179	91
		5'-TCTAACTGTTGGAGGTCTGGTC-3'		
Sycp3	NM_011517.2	5'-TTGCTGATGAAAAGGCTCCAGT-3'	261-357	97
		5'-TCCAGCATATTCTGTACTTCACCT-3'		
Tardbp	NM_145556.4	5'-GTCCGACTGGTGGAAGGAAT-3'	490-586	97
		5'-CCATTTTCCTTTTGTTATCTTTGGG-3'		

Table S2. Primers used for quantitative RT-qPCR for retinoic acid pathway targets. *Targets were obtained from Gely-Pernot *et al*. 2015 (Reference # 19)

Gene	**Accession number**	**Primers**	**Position (nucleotide)**	**Size (nucleotide)**
*RARgamma**	NM_011244.4	5’-CTCGGGTCTATAAGCCATGC-3’	746-805	60
		5’-CCCCATAGTGGTAGCCAGAA-3’		
*RXRalpha**	NM_001290481.1	5’-GATATCAAGCCGCCACTAGG-3’	384-534	151
		5’-TTGCAGCCCTCACAACTGTA-3’		
*RXRbeta**	NM_001205	5’-GGGCTGCAAGGGTTTCTTCA-3’	186-348	163
		5’-CTCCTGAACCGCCTCCCTTT-3’		
RBP4	NM_001159487.1	5’-GAGAGTTTGGCTCCACCGAG-3’	271-379	109
		5’-CCGGAGTCTGCCTCCTGAAC-3’		
Rdh11	NM_021557.6	5’-GTGGTGCTGAGATGTTCGGA-3’	55-148	94
		5’-CCACTGGACAGCATTTTCCTGA-3’		
Crbp1	NM_013496.3	5’-GGACGCAAATGCAGGAGTTT-3’	389-495	107
		5’-AGCTCTCGGGTCCAGTAAGT-3’		
Crbp2	NM_007759.2	5’-GATGGAGAGCTGATCCTGACAA-3’	497-602	106
		5’-GGAAGTCGTCTCAGGCAGTT-3’		
Rarres1	NM_001164763.1	5’-AGCATCAGCATACCTGACAGT-3’	595-683	89
		5’-ATACGTATGAGCTGCCGAG-3’		
Cyp26a1	NM_007811.2	5’-CAGATGGTGCTTCAGCGGAG-3’	277-392	116
		5’-ACATTATCCGCGCCCATCAC-3’		
*RARbeta**	NM_011243	5’-GCTGGGTCGTCGTTTTCTAA-3’	1167-1282	135
		5’-GAAACAGGCCTTCTCAGTGC-3’		
*RXRgamma**	NM_009107	5’-TGTGGTCAACAGTGTCAGCA-3’	6-190	185
		5’-AGAAGCCTTTGCAACCTTCA-3’		
*Sall4a**	NM_175303.4	5’-AGTGTCACCTGCCAATAGCC-3’	2560-2726	167
		5’-TGCCAGGCACTTCAACTTT-3’		
*Sall4b**	NM_201395.3	5’-CTCGACCAGTCCAAGAAAGG-3’	1232-1394	163
		5’-TGCCAGGCACTTCAACTTT-3’		
Stra6	NM_001162476.1	5’-TCTAGGAGCTGAGGAGGTGC-3’	474-583	110
		5’-GCAGAACCAGGAACGACAGT-3’		
